# Phylogenetic Diversity, Host-Specificity and Community Profiling of Sponge-Associated Bacteria in the Northern Gulf of Mexico

**DOI:** 10.1371/journal.pone.0026806

**Published:** 2011-11-02

**Authors:** Patrick M. Erwin, Julie B. Olson, Robert W. Thacker

**Affiliations:** 1 Department of Biology, University of Alabama at Birmingham, Birmingham, Alabama, United States of America; 2 Department of Biological Sciences, University of Alabama, Tuscaloosa, Alabama, United States of America; Université Paris Sud, France

## Abstract

**Background:**

Marine sponges can associate with abundant and diverse consortia of microbial symbionts. However, associated bacteria remain unexamined for the majority of host sponges and few studies use phylogenetic metrics to quantify symbiont community diversity. DNA fingerprinting techniques, such as terminal restriction fragment length polymorphisms (T-RFLP), might provide rapid profiling of these communities, but have not been explicitly compared to traditional methods.

**Methodology/Principal Findings:**

We investigated the bacterial communities associated with the marine sponges Hymeniacidon heliophila and Haliclona tubifera, a sympatric tunicate, Didemnum sp., and ambient seawater from the northern Gulf of Mexico by combining replicated clone libraries with T-RFLP analyses of 16S rRNA gene sequences. Clone libraries revealed that bacterial communities associated with the two sponges exhibited lower species richness and lower species diversity than seawater and tunicate assemblages, with differences in species composition among all four source groups. T-RFLP profiles clustered microbial communities by source; individual T-RFs were matched to the majority (80.6%) of clone library sequences, indicating that T-RFLP analysis can be used to rapidly profile these communities. Phylogenetic metrics of community diversity indicated that the two sponge-associated bacterial communities include dominant and host-specific bacterial lineages that are distinct from bacteria recovered from seawater, tunicates, and unrelated sponge hosts. In addition, a large proportion of the symbionts associated with H. heliophila were shared with distant, conspecific host populations in the southwestern Atlantic (Brazil).

**Conclusions/Significance:**

The low diversity and species-specific nature of bacterial communities associated with H. heliophila and H. tubifera represent a distinctly different pattern from other, reportedly universal, sponge-associated bacterial communities. Our replicated sampling strategy, which included samples that reflect the ambient environment, allowed us to differentiate resident symbionts from potentially transient or prey bacteria. Pairing replicated clone library construction with rapid community profiling via T-RFLP analyses will greatly facilitate future studies of sponge-microbe symbioses.

## Introduction

Sponge-microbe symbioses represent novel associations between an ecologically successful phylum of basal invertebrates and genetically diverse consortia of distinct microbial lineages [1]–[3]. Symbiotic bacterial communities often exhibit high abundance within the sponge host, comprising up to 35% of total holobiont biomass [4], while the biodiversity of sponge-associated microorganisms includes representatives from most major clades of Bacteria [5], [6] and Archaea [7]–[9]. In fact, recent deep sequencing of sponge microbiota revealed the highest diversity of bacterial symbionts for any invertebrate host investigated to date [10]. A multitude of metabolic functions underlies this extensive diversity, including nitrification [9], [11]–[13], denitrification [14], nitrogen fixation [15], [16], sulfur oxidation [17], and carbon fixation [18]–[21]. Symbiotic microbial communities can significantly impact host sponge ecology and evolution through the provision of supplemental nutrition [21]–[25] and the production of secondary metabolites [26] that deter predators, competitors and fouling organisms [27].

The broad implications of sponge-bacterial symbioses have prompted a recent surge in the field of sponge microbiology [1]–[3], but many fundamental questions remain unresolved. For example, it is often unclear whether these symbionts are generalists that associate with all sponges at a particular location, or specialists that associate with a single host species. In addition to mutualistic symbionts, bacteria recovered from sponges may also represent: 1) a food source that is selectively filtered and consumed, 2) parasitic microbes acting as invasive pathogens [28], [29], 3) fouling species [30], [31], or 4) transient microorganisms in the ambient environment at the time of sample collection. Numerous comparisons of sponge-derived microbes to environmental bacteria using culture-dependent and culture-independent (i.e., molecular) techniques have reported clear distinctions between sponge-associated microbes and ambient sediment [32] and seawater bacteria [5], [8], [10], [32]–[38].

Molecular evidence initially revealed 14 sponge-associated bacterial clades that are absent from seawater bacterial communities [5]. These phylogenetically diverse and sponge-specific clades inhabit taxonomically diverse host species from geographically distant regions [5], [39], [40] and are hypothesized to represent a “universal” bacterial community within sponge hosts. An extensive phylogenetic analysis of over 1,500 sponge-derived bacterial 16S rRNA gene sequences available in the GenBank database showed that nearly one-third (32%) of all sponge-associated bacteria fall into monophyletic, sponge-specific clusters [2]. Other studies suggest an even higher degree of host-specificity between sponges and bacteria, with distinct symbiont 16S rRNA phylotypes consistently associated with particular host species [8], [32], [35], [41]–[44] or genera [45] and some molecular data supporting potential host-symbiont coevolution [46], [47].

Investigations of stability and fluctuations in sponge-bacteria symbioses, in conjunction with on-going studies of diversity, have begun to assess the dynamics of host-symbiont relationships [35], [41], [48]–[51] and the factors that may disrupt the symbiosis, such as pollutants [52], thermal stress [53], [54], and disease outbreaks [13], [55]. Such studies typically involve large sample sizes and employ DNA fingerprinting techniques to rapidly profile symbiont communities, since traditional clone library construction and DNA sequencing become increasingly laborious and expensive with larger sets of samples [56]. In particular, denaturing gel gradient electrophoresis (DGGE) analyses have been prominent in the study of sponge microbiology [8], [35], [40], [41], [43], [48], [51], [57]–[60]. In addition, a few studies have used terminal restriction fragment length polymorphism (T-RFLP) analyses to monitor surface-fouling communities [31] and archaeal [13], [61] and bacterial symbionts [43], [51], [62]. General patterns of microbial community profiles are often similar between DGGE and T-RFLP [43], [51], although increased reproducibility and resolution has been observed with T-RFLP analyses compared to DGGE analyses [43], [51], likely due to the standardization of T-RFLP analyses via an automated capillary electrophoresis platform. In fact, T-RFLP analysis revealed similar community-level patterns as massively parallel pyrosequencing in Red Sea sponges [62].

Accurate characterization of sponge-associated microbial communities is an essential step in resolving sponge-microbe interactions and understanding the importance of these symbiotic assemblages to their host sponges. The patterns of host-specificity and community structure revealed to date are derived from a relatively small number of host species, compared to extant sponge biodiversity (over 8,000 species [63]); therefore, further study of additional sponges from varying geographical regions is required to understand the prevalence and ecological implications of hosting specialist and generalist symbiont communities. Moreover, since most studies of sponge-symbiont associations to date lack sufficient replication for rigorous statistical analyses of host-specificity, we sought to demonstrate the utility of a replicated sampling strategy.

The sponge *Hymeniacidon heliophila*, commonly termed the “sun sponge,” inhabits shallow-water, near-shore environments throughout the western Atlantic, Gulf of Mexico and Caribbean [64], including intertidal zones [65] and artificial substrates [66]. *H. heliophila* also colonizes pilings of offshore oil and natural gas drilling platforms in the northern Gulf of Mexico (this study) and appears to represent a pollution-tolerant species able to adapt to eutrophic environmental conditions [67]. The local abundance and widespread distribution of *H. heliophila* from high-impact coastal zones and artificial substrata to natural reef environments renders this species ideal for the study of biogeography, holobiont fitness, symbiont dynamics and disturbance responses in sponge-microbial symbioses.

The associated bacterial and archaeal communities of *H. heliophila* have been investigated for host populations in the southwestern Atlantic [32], [67], but remain unknown for most of the species' geographic range. In this study, we investigated the bacterial community associated with *H. heliophila* from the northern Gulf of Mexico, along with the communities associated with a sympatric sponge, *Haliclona tubifera*, a sympatric tunicate, *Didemnum* sp., and the ambient seawater. The inclusion of a distantly related sponge host and a non-sponge host from the same location as the focal species, *H. heliophila*, allowed us to statistically test whether unique taxa were found in each host or whether these hosts share a common microbial community derived from the surrounding seawater. Our study entailed three specific aims: (1) to characterize and compare the community structure, diversity and specificity of these microbial communities using replicated 16S ribosomal RNA (rRNA) gene sequence libraries and phylogenetic metrics of community diversity, (2) to assess the ability of T-RFLP analyses to rapidly profile these microbial communities and the congruence between T-RFLP and 16S rRNA gene sequence data, and (3) to compare the diversity and specificity of microbial communities from *H. heliophila* in the Gulf of Mexico to populations in the southwestern Atlantic and other sponge-associated communities.

## Results

### Diversity and composition of microbial communities

**Figure 1:**
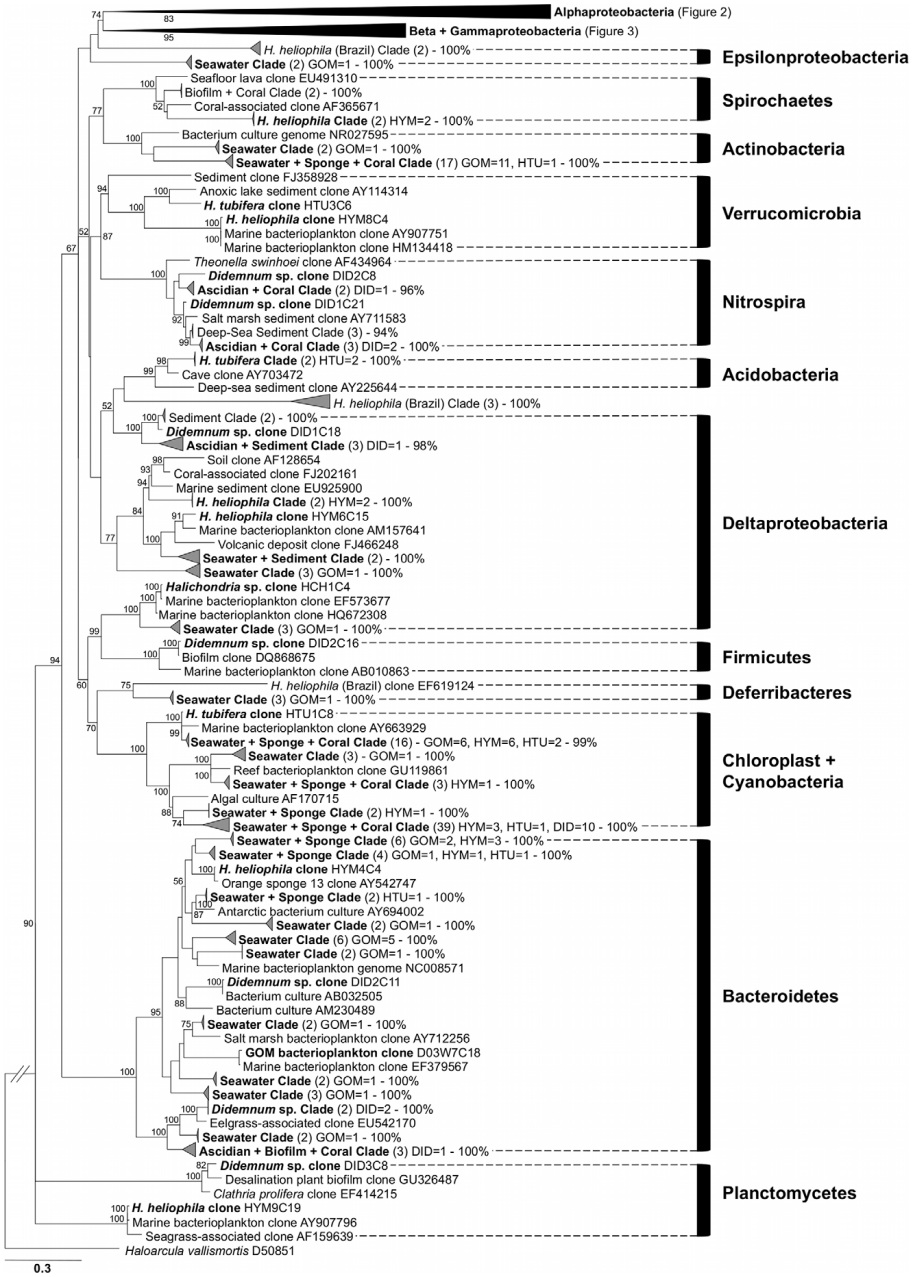
Phylogeny of bacterial 16S rRNA gene sequences recovered from sponges, tunicates and seawater. Terminal node labels denote the sequence source and GenBank accession number; for condensed clades (gray triangles) the total number of sequences (in parentheses), sequences from this study (abbreviations) and bootstrap support (%) for each clade are shown. Bold labels highlight individual sequences or clades containing sequences from in this study. Tree topology was constructed using maximum likelihood criteria and numbers on nodes depict bootstrap support (100 replicates; values <50% not shown). Condensed clades for *Alphaproteobacteria* and *Betaproteobacteria*+*Gammaproteobacteria* are expanded in Figure 2 and Figure 3, respectively. The full phylogeny is available as supplemental material (Figure S1). GOM = Gulf of Mexico seawater, HYM = *Hymeniacidon heliophila*, HTU = *Haliclona tubifera*, HCH = *Halichondria* sp. and DID = *Didemnum* sp.

**Figure 2:**
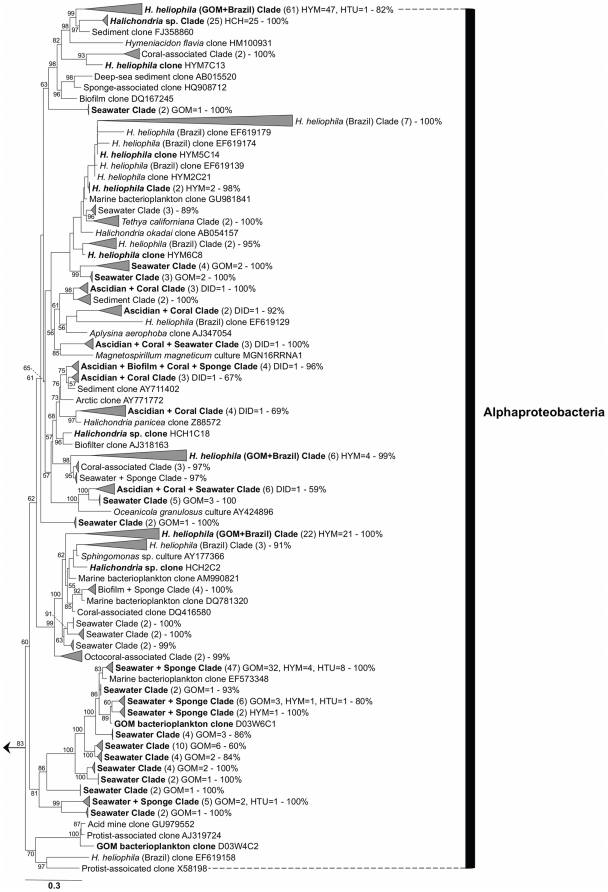
Phylogeny of bacterial 16S rRNA gene sequences recovered from sponges, tunicates and seawater: *Alphaproteobacteria*. Labels and abbreviations as in Figure 1. The full phylogeny is available as supplemental material (Figure S1).

**Figure 3:**
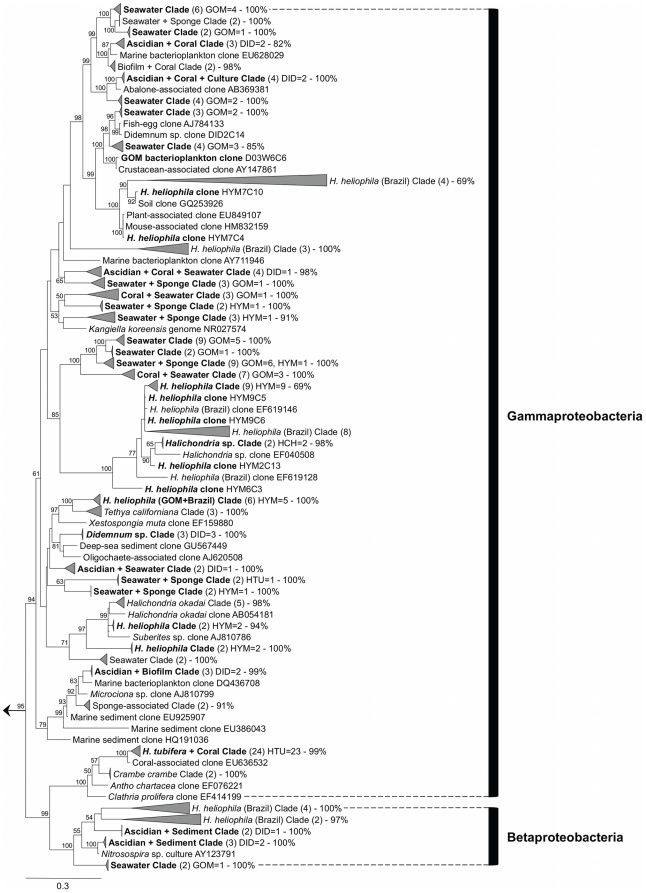
Phylogeny of bacterial 16S rRNA gene sequences recovered from sponges, tunicates and seawater: *Betaproteobacteria* and *Gammaproteobacteria*. Labels and abbreviations as in Figure 1. The full phylogeny is available as supplemental material (Figure S1).

A total of 389 bacterial sequences were recovered from sponge, tunicate, and seawater samples, representing 159 unique bacterial operational taxonomic units (OTUs), according to an OTU definition of 99% similarity (Figure 1, Figure 2, Figure 3, Figure S1). These sequences were deposited in GenBank as accession numbers EU315321-EU315680 and JF824738-JF824766 (Table S1). The combined clone library was dominated by 4 OTUs that accounted for 36.7% of all clones and corresponded to 3 representatives of *Alphaproteobacteria* (Figure 2) and 1 of *Gammaproteobacteria* (Figure 3). Recovered sequences spanned 13 bacterial lineages, with *Alphaproteobacteria* accounting for nearly half (46.9%) of all screened clones (Table 1, Table S1). Other common lineages included *Gammaproteobacteria*, *Cyanobacteria*, *Bacteroidetes*, and *Actinobacteria*, together accounting for an additional 45.6% of all clones recovered (Table 1, Table S1). Several lineages were recovered solely from one source, including *Acidobacteria* in *Haliclona tubifera* and *Nitrospira* in *Didemnum* sp. (Table 1, Figure 1, Figure S1). Bacterial communities exhibited very little overlap in OTU composition across sources. The vast majority of bacterial sequences (92.8%) were recovered exclusively from one source. Furthermore, different proportions of major taxonomic groups were recovered from each source (Table 1; *G* = 63.5, *df* = 9, *P*<0.001). *Hymeniacidon heliophila* was associated with a greater proportion of *Alphaproteobacteria* than expected by chance, while *H. tubifera* had a much larger proportion of *Gammaproteobacteria* than observed in any other group. A third sponge, *Halichondria* sp. was only collected twice; its bacterial community was dominated by *Alphaproteobacteria* (Table S1, Figure 2), but due to the lack of sufficient replicate samples, this species was excluded from subsequent statistical and T-RFLP analyses. *Cyanobacteria* were over-represented in *Didemnum* compared to the other sources, while seawater and tunicate samples included a greater proportion of *Bacteroidetes* and rare taxa than sponge samples (Table 1).

**Table 1:** Major bacterial divisions represented in sponge, tunicate and seawater bacterial communities, shown as percentages of the total recovered community.

	*H. heliophila*	*H. tubifera*	*Didemnum* sp.	Seawater
	(*n* = 135)	(*n* = 45)	(*n* = 45)	(*n* = 135)
*Alphaproteobacteria*	**63.0**	**24.4**	**15.6**	**48.9**
*Gammaproteobacteria*	**20.0**	**53.3**	**26.7**	**22.2**
*Cyanobacteria*	8.1	8.9	**22.2**	5.2
*Bacteroidetes*	3.7	4.4	8.9	**11.1**
*Actinobacteria*	-	2.2	-	8.9
*Deltaproteobacteria*	2.2	-	4.4	1.5
*Nitrospira*	-	-	11.1	-
*Betaproteobacteria*	-	-	6.7	0.7
*Verrucomicrobia*	0.7	2.2	-	-
*Planctomycetes*	0.7	-	2.2	-
*Spirochaetes*	1.5	-	-	-
*Acidobacteria*	-	4.4	-	-
*Firmicutes*	-	-	2.2	-
*Epsilonproteobacteria*	-	-	-	0.7
*Deferribacteres*	-	-	-	0.7

Numbers in parentheses refer to total clones recovered for each source. Bold values represent dominant lineages associated with each source.

The *H. heliophila* bacterial community exhibited the second highest number of unique OTUs (*n* = 37) and was comprised mostly of *Alphaproteobacteria* (63.0%; Table 1, Figure 2) and *Gammaproteobacteria* (20.0%; Table 1, Figure 3). Chao1 estimation predicted that 75 OTUs were present in the *H. heliophila* bacterial community, with the observed OTUs accounting for 49.3% of the total community. A single dominant specialist *Alphaproteobacteria* symbiont was recovered, accounting for over one-third (34.1%) of all clones and present in all samples of *H. heliophila*. Six OTUs (34.1% of clones) represented common specialist symbionts, 11 OTUs (10.4%) were rare specialist symbionts, and the remaining 19 OTUs (21.5%) were classified as generalist symbionts. Singleton OTUs, those occurring only once in the clone library, accounted for the majority (*n* = 23, 62.6%) of recovered OTUs, with most singleton OTUs (*n* = 15, 65.2%) closely related to free-living bacteria (Table S1).

The *Haliclona tubifera* bacterial community exhibited the lowest number of unique OTUs (*n* = 14), and was comprised mostly of *Gammaproteobacteria* (53.3%; Table 1, Figure 3) and *Alphaproteobacteria* (24.4%; Table 1, Figure 2). Chao1 estimation predicted that 30 OTUs were present in the *H. tubifera* bacterial community, with the observed OTUs accounting for 46.6% of the total community. A single dominant specialist *Gammaproteobacteria* symbiont was recovered, accounting for over half (51.1%) of all clones and present in all samples (Figure 3). Three OTUs (8.9% of clones) represented rare specialist symbionts, while the remaining 10 OTUs (40.0% of clones) were classified as generalist symbionts. Singleton OTUs accounted for the majority (*n* = 10, 71.4%) of recovered OTUs, with most singleton OTUs (*n* = 8, 80.0%) closely related to free-living bacteria (Table S1).

The seawater bacterial community exhibited the highest number of unique bacterial OTUs (*n* = 65) and, similar to the *H. heliophila* and *H. tubifera* bacterial communities, was comprised mostly of *Alphaproteobacteria* (48.9%; Table 1, Figure 2) and *Gammaproteobacteria* (20.0%; Table 1, Figure 3). Chao1 diversity estimation predicted that 155 OTUs were present in this seawater community, with the observed OTUs accounting for 41.9% of the total community. A single *Alphaproteobacteria* OTU dominated the seawater bacteria; this OTU accounted for 23.0% of the clone library and was recovered from all 9 samples (Figure 2). Another 16 OTUs (39.3% of clones) were common, recovered from more than one sample, and 48 OTUs (37.8%) were rare, recovered from a single seawater sample. Singleton OTUs (*n* = 45, 69.2%) accounted for the majority of bacterial OTUs derived from seawater (Table S1).

The *Didemnum* sp. bacterial community exhibited 35 unique OTUs, similar to the *H. heliophila*-associated community, despite two-thirds fewer clones screened. The *Didemnum* community was comprised mostly of *Gammaproteobacteria* (28.9%; Table 1, Figure 3) and *Cyanobacteria* (22.2%; Table 1, Figure 1). Chao1 estimation predicted 81 OTUs in the *Didemnum* sp. bacterial community, with the observed OTUs accounting for 43.2% of the estimated OTU richness. The *Didemnum*-associated bacteria displayed a more even community, with no dominant OTUs present and no OTUs recovered from all samples. One OTU (6.7% of clones) represented a common specialist symbiont, isolated from 2 of 3 *Didemnum* sp. samples. Sixteen OTUs (40.0% of clones) represented rare specialist symbionts, while the remaining 18 OTUs (53.3% of clones) were classified as generalist symbionts. Singleton OTUs accounted for the majority (*n* = 27, 77.1%) of recovered OTUs, with less than half of singleton OTUs (*n* = 13, 48.1%) closely related to free-living bacteria (Table S1).

### Comparative analysis of microbial communities

**Figure 4:**
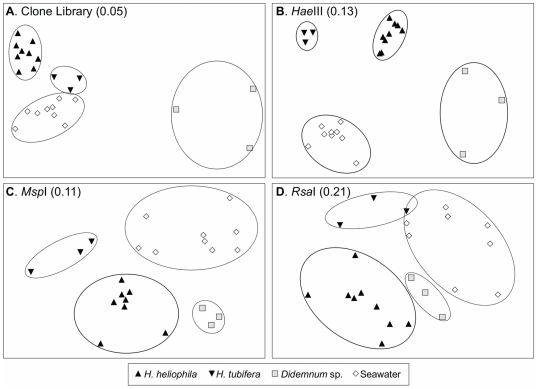
Host-specificity of bacterial communities associated with sponge, tunicate and seawater samples. Non-metric multi-dimension scaling (MDS) plots of bacterial communities recovered from sponge, tunicate and ambient seawater samples constructed from 16S rRNA gene sequence libraries (A) and T-RFLP profiles with *Hae*III (B), *Msp*I (C) and *Rsa*I (D). Circles encompass all samples from each source and highlight the distinct nature of bacterial communities from each source. Stress values are shown in parenthesis and values below 0.15 indicate an excellent match between MDS ordination distances and similarity matrix distances.

Both traditional ecological metrics and molecular phylogenetic metrics revealed significant differences among the bacterial communities associated with different sources. The seawater and tunicate-associated bacterial communities were significantly more species-rich and diverse than sponge-associated bacterial communities, in terms of observed species richness (ANOVA, *P*<0.05; Table 2), Shannon diversity index (ANOVA, *P*<0.05; Table 2), and rate of unique OTU accumulation (ANCOVA, *P*<0.05; Figure S2). In addition to differences in OTU richness and diversity, the community structure of *H. heliophila* associated bacteria (relative abundances and presence-absence of OTUs) differed significantly from those of the bacterial communities in ambient seawater, the sympatric sponge *H. tubifera* and the sympatric tunicate *Didemnum* sp. (ANOSIM, *P*<0.05; Table S2, Figure 4). The community structure of *H. tubifera* associated bacteria also differed significantly from ambient seawater bacterial assemblages when considering relative abundance of OTUs (ANOSIM, *P*<0.05; Table S2, Figure 4) but not when considering the presence-absence of OTUs (ANOSIM, *P* = 0.06; Table S2), indicating that the observed difference was due to the high relative abundance of a single unique phylotype of *Gammaproteobacteria*. In addition, the bacterial communities in *H. tubifera* were not significantly dissimilar to those recovered from the tunicate *Didemnum* sp. (ANOSIM, *P* = 0.10, Table S2, Figure 4).

**Table 2:** Average species richness (S_obs_), Shannon diversity (H′) and evenness (J) indices for bacterial communities recovered from sponge, tunicate and seawater samples.

	No.	S_obs_	H′	J
*H. heliophila*	9	7.44 (±0.41)^a^	1.76 (±0.08)^a^	0.88 (±0.02)^a^
*H. tubifera*	3	6.33 (±0.33)^a^	1.44 (±0.03)^a^	0.76 (±0.02)^b^
Seawater	9	11.56 (±0.71)^b^	2.29 (±0.10)^b^	0.94 (±0.02)^a^
*Didemnum* sp.	3	12.33 (±0.33)^b^	2.45 (±0.04)^b^	0.98 (±0.01)^a^

Numbers in parentheses correspond to ±1 SE. Superscript letters denote differences among sources.

Phylogenetic diversity analyses confirmed the presence of significant differences among the four bacterial communities (LIBSHUFF, *P*<0.001 among sources and all pairwise comparisons; AMOVA, *P*<0.001 among sources; Table S3), with *Hymeniacidon*, *Didemnum*, and seawater harboring distinct phylogenetic lineages of bacteria (P-test, *P*<0.005 when comparing these three sources, Table S3) but not *H. tubifera* (P-test, *P*>0.23, Table S3). Both sponge-associated bacterial communities were significantly clumped, or phylogenetically under-dispersed (Table S4), likely a result of the unique lineages of bacterial phylotypes that dominated these assemblages.

Bacterial communities were similar among collection locations (i.e., drilling platforms). An ANOSIM conducted with collection location as a factor revealed no significant differences (*P* = 0.33) among platforms. Likewise, comparisons of average species richness, the Shannon index, evenness, and the Chao1 estimator were not significantly different among platforms (all *P*>0.14). Although an AMOVA comparing platforms found no significant variation (*F_ST_* = 0.010, *P* = 0.937), a P-test revealed significant lineage sorting among locations (*P*<0.001), even when seawater samples were excluded. This pattern could be created by the strong influence of water column bacteria on the community associated with *H. tubifera*, because while unique lineages were present at each location, the total amount of genetic variation did not differ among locations.

**Figure 5:**
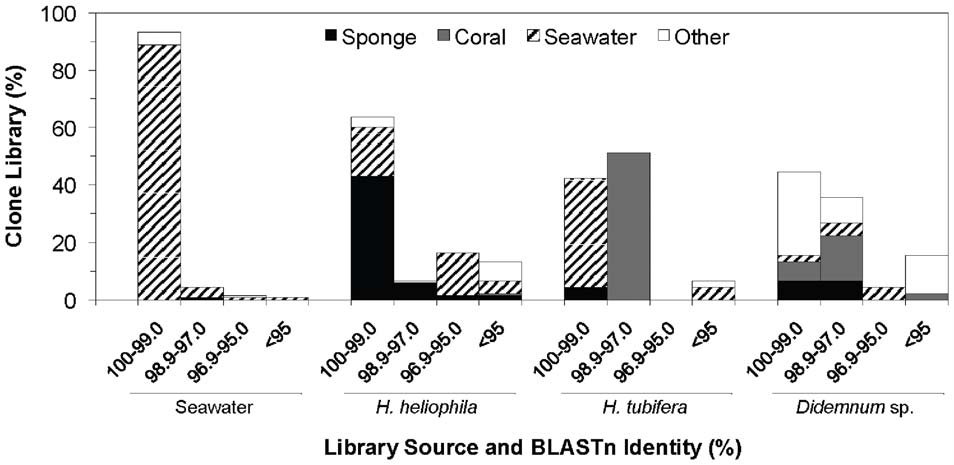
Similarity of bacterial sequences recovered from sponge, tunicate and seawater samples to GenBank sequences. Results from BLAST searches are grouped by sequence identity and highlight the source of each GenBank sequence.

Comparison of recovered bacterial sequences to the GenBank database revealed a unique pattern of affiliation with previously reported sources of bacteria in each of the four bacterial communities (*G* = 623.1, *df* = 9, *P*<0.001; Figure 5). The vast majority (89%) of seawater clones were closely related (≥99% identity) to other bacterioplankton-derived sequences (Figure 5). Sponge-associated bacterial communities exhibited some overlap with seawater microbes, with 17.0% (*H. heliophila*) and 37.8% (*H. tubifera*) of clones matching closely (≥99% identity) to bacterioplankton sequences; however, the majority of clones from these libraries matched to other invertebrate-derived sequences or were distantly related (<97% identity) to seawater bacteria. In *H. heliophila*, over half of all clones (*n* = 68, 50.4%) matched to sequences derived from *H. heliophila* in Brazil (Turque et al. 2008), with the majority of these clones (*n* = 58, 85.3%) exhibiting nearly identical sequence similarity (≥99%). In contrast, bacterial clones from *H. tubifera* only rarely matched to other sponge-derived sequences (*n* = 2, 4.4%). Rather, these communities were most commonly matched to coral-derived sequences (*n* = 25, 55.5%), due largely to the close relationship between the single dominant symbiont in *H. tubifera* and coral-associated bacteria. Bacterial clones recovered from *Didemnum* sp. matched to sequences derived from a variety of sources, with only 1 singleton OTU matching to another ascidian-derived sequence (GenBank accession number DQ860071 [68]). Other clones from *Didemnum* sp. were related to bacteria derived from the sponge *Tethya californiana* (*n* = 6, 13.3% of clones [69]), various coral species (*n* = 11, 24.4%) and marine sediment (*n* = 9, 20%).

### Phylogenetic analysis of microbial communities

Phylogenetic analysis revealed that in *H. heliophila* bacterial communities, several symbiont lineages were present in hosts from both the Gulf of Mexico and the southwestern Atlantic, forming shared monophyletic clades or closely related sister taxa (Figure 1, Figure 2, Figure 3). In particular, 4 well-supported monophyletic clades were recovered that were comprised solely of sequences derived from *H. heliophila* (Figure 1, Figure 2, Figure 3; “GOM+Brazil” clades), including the dominant *Alphaproteobacteria*-affiliated phylotype in *H. heliophila* from the Gulf of Mexico that also exhibited high relative abundance (*n* = 13, 20.6% of clones) in *H. heliophila* from Brazil [32]. Notably, related bacterial sequences were recovered from sympatric *Halichondria* sp. samples in the Gulf of Mexico; these sequences formed the dominant symbiont phylotypes of this host (*n* = 25, 83.3% of clones). Other *H. heliophila*-derived symbiont clades were affiliated with *Alphaproteobacteria* (*n* = 2; Figure 2) and *Gammaproteobacteria* (*n* = 1; Figure 3). A final sequence cluster was affiliated with *Gammaproteobacteria* (Figure 3) and contained clones derived from *Halichondria* spp. (*n* = 3) in addition to *H. heliophila* from the Gulf of Mexico (*n* = 12) and Brazil (*n* = 10). In total, these shared symbiont clades accounted for 65.2% and 42.9% of bacterial sequences derived from *H. heliophila* in the Gulf of Mexico and Brazil, respectively. The remaining clones, specific to a single geographic region, were affiliated with diverse bacterial phyla (e.g., *Bacteroidetes*, *Deferribacteres* and others) and closely matched seawater bacterioplankton (e.g., *Planctomycetes* and *Verrucomicrobia*) or formed distinct symbiont clades specific to one host population (e.g., *Spirochaetes* and *Deltaproteobacteria* clones in *H. heliophila* from the Gulf of Mexico).

Phylogenetic analysis also revealed no overlap between the sponge-associated bacterial communities recovered in this study and previously described sponge-specific lineages [2], [5]. A single sequence was recovered from BLAST searches that corresponded to a sponge-specific symbiont lineage: a *Nitrospira*-affiliated clone from *Theonella swinhoei* (AF434964 [5]) that matched most closely (89.5% sequence identity) to a *Didemnum* sp. derived sequence and grouped separately in the phylogenetic tree (Figure 1).

Similar to BLAST search results, the phylogenetic analysis of bacteria associated with *Didemnum* sp. revealed that these communities overlapped with numerous other sources, including bacterioplankton, sediment, biofilms, sponges and corals. Most strikingly, several symbionts derived from the ascidian were shared with numerous coral species and formed distinct sequence clusters in phylogenetic trees. These occurred most prominently in clones affiliated with *Alphaproteobacteria*, where 4 monophyletic “Ascidian+Coral” clades were recovered (Figure 2). Within the *Gammaproteobacteria*, another symbiont clade specific to ascidians and corals was reported, along with the single *Didemnum* sp. specific clade (*n* = 3 sequences) recovered (Figure 3). In addition, two monophyletic clades of *Nitrospira*-affiliated sequences from *Didemnum* sp. and coral hosts occurred, forming a larger sequence cluster with various sediment-derived clones (Figure 1).

### T-RFLP analysis

A total of 120 T-RFs were identified from T-RFLP profiles analyzed using the restriction enzyme *Hae*III (45 in *H. heliophila*, 32 in *H. tubifera*, 44 in *Didemnum* sp. and 50 in seawater); 65 T-RFs were identified with *Msp*I (22 in *H. heliophila*, 14 in *H. tubifera*, 18 in *Didemnum* sp. and 32 in seawater); and 62 T-RFs were identified with *Rsa*I (36 in *H. heliophila*, 22 in *H. tubifera*, 16 in *Didemnum* sp. and 21 in seawater). The majority of recovered T-RFs were isolated solely from a single source (sponge, tunicate or seawater): 65.9% in *Hae*III digests, 78.5% in *Msp*I digests and 61.3% in *Rsa*I digests. No T-RFs were present in all sources. Average bacterial diversity (number of T-RFs per profile) varied significantly among restriction enzymes (*P*<0.001), with T-RFLP analysis using *Hae*III revealing twice as much diversity as either *Msp*I or *Rsa*I across all sources (Figure S3). No significant differences in diversity were observed based on source (*P* = 0.128) and no significant interaction occurred between source and restriction enzyme (*P* = 0.318).

T-RFLP profiles produced distinctive microbial community fingerprints based on isolation source (*H. heliophila*, *H. tubifera*, *Didemnum* sp. or seawater; Figure S4). Non-metric multi-dimensional scaling (MDS) plots exhibited discrete clustering of samples based on source, indicating clear distinctions between the bacterial communities, and were consistent across different analysis methods (relative abundance, presence-absence) and restriction endonucleases (Table S2, Figure 4). Similar to results from 16S rRNA gene sequence clone libraries, the relative abundances and presence-absence of bacterial T-RFs associated with *H. heliophila* were significantly different from those in ambient seawater, the sympatric sponge *H. tubifera* and the sympatric tunicate *Didemnum* sp. (ANOSIM, *P*<0.05; Table S2). *H. tubifera* bacterial community structure was also significantly different from ambient seawater bacterial assemblages for most analysis methods and restriction endonucleases (ANOSIM, *P*<0.05), with the single exception of presence-absence data from *Rsa*I (ANOSIM, *P* = 0.06; Table S2). In addition, the bacterial communities in *H. tubifera* were not significantly dissimilar from the tunicate *Didemnum* sp. for any data analysis and restriction endonuclease combination (ANOSIM, *P* = 0.10, Table S2). MDS plots constructed from T-RFLP analysis using *Hae*III, the enzyme which displayed the highest number of distinct T-RFs (Figure S3), showed the clearest distinctions among bacterial communities and exhibited discrete and tight sample clusters based on isolation source and high R-statistic values (Table S2; Figure 4).

### Congruence between T-RFLP and clone library analysis

*In silico* digestion of our clone library sequences predicted that T-RFLP analysis using *Hae*III would match the highest number and percentage of clone sequences (*n* = 100, 71.9%), followed by *Msp*I (*n* = 98, 70.5%) and *Rsa*I (*n* = 63, 45.3%). Together, the enzymes were predicted to account for 133 (95.7%) of the clone library sequences. Empirical T-RFLP analysis corroborated *in silico* predictions, with peak profiles from the *Hae*III digestion matching to the highest number and percentage of clones (*n* = 71, 51.5%), followed accordingly by *Msp*I (*n* = 57, 41.0%) and *Rsa*I (*n* = 40, 29.0%; Table S5). Together, the 3 enzymes accounted for 112 (80.6%) of the clones from 16S rRNA gene sequence libraries (Table S5). The frequency of major taxonomic groups of bacteria recovered using T-RFLP did not differ significantly from clone library analysis for the enzyme *Hae*III (*G* = 11.4, *df* = 12, *P* = 0.49) and the combination of all enzymes (*G* = 11.7, *df* = 12, *P* = 0.47); however, significant differences were observed for the enzymes *Msp*I (*G* = 28.3, *df* = 12, *P*<0.05) and RsaI (*G* = 28.1, *df* = 12, *P*<0.05). The bacterial community recovered using T-RFLP analysis with *Msp*I exhibited a greater proportion of *Alphaproteobacteria* and *Gammaproteobacteria* than expected from clone library analysis and a lower proportion of *Bacteroidetes*, while T-RFLP analysis with *Rsa*I exhibited a greater proportion of *Bacteroidetes*.

While the vast majority of clone library sequences were represented in T-RFLP profiles, less than half (*n* = 57, 41.0%) of distinct phylotypes presented unique T-RFLP signatures. An additional 55 phylotypes (39.6%) presented shared T-RFLP signatures and were thus detected in T-RFLP profiles but not individually distinguishable. The remaining 27 phylotypes (19.4%) presented no T-RFs within the detected range (100–500 bp) for any of the 3 enzymes and thus were not recovered in T-RFLP profiles.

The resolution of empirical T-RFLP signatures varied depending on the number of enzymes represented within each signature. When 1 or 2 enzymes were represented, approximately half (*n* = 52, 48.6%) of the T-RFLP signatures were unique, while the remaining (*n* = 55, 51.4%) were shared between at least 2 distinct phylotypes. In the few cases where all 3 enzymes were represented (*n* = 5), all T-RFLP signatures were unique. Notably, most phylotypes that produced shared T-RFLP signatures represented related bacteria, averaging 5.8% sequence divergence (±0.95 SE). Among the distinct phylotypes that exhibited shared T-RFLP signatures, those with 2 enzymes represented (*n* = 22) were more closely related (3.1%±1.0 sequence divergence) than those with only 1 enzyme represented (*n* = 33; 7.7%±1.3 sequence divergence). No more than 3 phylotypes matched to a single T-RFLP profile (average ± SE = 2.2±0.1).

Clone libraries accounted for over half (*n* = 127, 51.4%) of recovered T-RFs, including 56 matches from *Hae*III profiles (46.7% of total T-RFs, Table S6), 40 matches from *Msp*I (61.5% of total T-RFs, Table S7) and 31 matches from *Rsa*I (50.0% of total T-RFs, Table S8). In many cases, specific ranges of T-RF length were consistently matched to one bacterial lineage. For example, 435–449 bp T-RFs in *Msp*I profiles matched to 22 *Alphaproteobacteria* sequences (Table S7) and 313–318 bp T-RFs in *Rsa*I profiles matched to 13 *Bacteroidetes* sequences (Table S8). In other cases, unrelated bacteria shared terminal cut sites and T-RFs. For example, 228–230 bp T-RFs in *Hae*III profiles matched to 16 sequences representing *Alphaproteobacteria*, *Betaproteobacteria* and *Cyanobacteria* (Table S6).

## Discussion

The bacterial communities associated with the marine sponges *H. heliophila* and *H. tubifera* were differentiated from the bacterial communities associated with sympatric tunicates and seawater, exhibiting lower species richness, lower species diversity and host-specific bacterial phylotypes. These results lend further support to the hypothesis that sponges host unique microbial assemblages that are distinct from the microbial community found in ambient seawater. Additionally, differentiation of sponge-associated and tunicate-associated bacteria suggests that the recovered bacteria do not represent members of a generalist fouling community, contamination from marine sediment or shared bacterioplankton prey. Each host sponge species harbored a unique bacterial assemblage and shared only 4 bacterial OTUs, with 2 of these OTUs also present in ambient bacterioplankton communities. Notably, none of the recovered sequences belonged to the previously described sponge-specific clades [2], [5], suggesting that specialized and host-specific bacterial symbionts inhabit the temperate sponges *H. heliophila* and *H. tubifera*.

The low diversity and species-specific nature of bacterial symbionts in *H. heliophila* and *H. tubifera* represent a distinct form of sponge-bacteria symbiosis that appears to be quite different from the commonly reported “universal bacterial community” of other marine sponges [2], [5]. Previous studies have also reported specialist sponge-associated bacterial communities, distinct from seawater bacteria and the microflora of sympatric sponge species [8], [32], [35], [41]–[43]. An interesting, yet preliminary trend is that sponges hosting specialist microbial communities tend to represent low-microbial-abundance (LMA) species (e.g., *Mycale laxissima* [70] and *Ianthella basta* [71]), while high-microbial-abundance (HMA) sponges often host generalist lineages of symbionts [70]. Consistent with this hypothesis, electron microscopy data suggest that *H. heliophila* is a LMA sponge [32]. Future studies incorporating microbial abundance data and phylogenetic analyses are needed to test for potential correlations between symbiont abundance and symbiont specificity.

A prominent feature of the bacterial communities associated with *H. heliophila* and *H. tubifera*, as well as *Halichondria* sp., was the presence of one or few dominant and species-specific symbionts associated with each host. For example, a single specialist phylotype dominated the *H. tubifera* community, with the remainder of the community found predominately in seawater. Dominance of symbiotic communities by a small number of phylotypes has also been observed in *Ianthella basta*, where two phylotypes accounted for >90% of all clone library sequences [71] and a single OTU (at a 97% sequence similarity definition) comprised nearly half (49%) of all recovered high-throughput V6 16S rRNA sequences [10]. Further, over one-fifth of the sequences recovered from *H. heliophila* in Brazil (*n* = 13, 20.6% of clones [32]) formed a monophyletic group with the dominant phylotype in *H. heliophila* presented herein. Several hypotheses concerning the maintenance (e.g., vertical transmission) and implications (e.g., competitive exclusion of other microbes) of dominant bacterial symbionts are tempting from these observed trends; however, the relative abundance of bacteria in clone libraries must be interpreted with caution, due to the potential for selective PCR-amplification and over-representation of specific phylotypes. Additional data from microscopy and fluorescent *in situ* hybridization (FISH) [72]–[74] are required to fully test these hypotheses.

Comparison of the bacterial community in *H. heliophila* from the northern Gulf of Mexico and southwestern Atlantic revealed several striking similarities and notable differences in symbiont diversity and structure between these distant, conspecific host populations. *H. heliophila* sponges in Brazil harbored higher diversity communities (S_Chao1_ = 230) than sponges in the Gulf of Mexico (S_Chao1_ = 75). Although fewer clones were screened (*n* = 66) and a more conservative OTU definition (97% sequence identity) was employed by Turque et al. [32] for *H. heliophila* in Brazil, these differences would only decrease their diversity estimates compared to the analyses herein, thus making the recovered trend particularly noteworthy. From a broad taxonomic view, both host sponge populations exhibited a high prevalence of *Alphaproteobacteria* and *Gammaproteobacteria*, but differed in the number and composition of rare bacterial phyla. Several of the phylotypes affiliated with *Alpha*- and *Gammaproteobacteria* were shared among the biogeographically separate hosts, found to be exclusive to this species, and accounted for a large portion (>40%) of each symbiont community. The presence of shared bacterial phylotypes in distant populations of *H. heliophila* suggests a high potential for host-specificity in these symbiont lineages, which should be further investigated in adults and larvae using targeted FISH.

Two phylotypes associated with *H. heliophila* also formed larger sequence clusters with clones derived from closely related host sponges in the family Halichondriidae. An *Alphaproteobacteria*-affiliated cluster that contained the dominant phylotype recovered in *H. heliophila*, also included symbiont clones harbored by the congeneric species *H. sinapium* from Japan (HM100889) and *H. flavia* from Korea (HM100931), as well as 2 *Halichondria* spp. from the Gulf of Mexico (this study) and Korea (EF040530). *Gammaproteobacteria*-affiliated sequences from the same 2 *Halichondria* spp. formed a second sequence cluster with *H. heliophila* clones from the Gulf of Mexico and Brazil (Figure 3). Other sequences grouping within these clusters were recovered from non-sponge sources, including other invertebrate hosts (coral species *Porites compressa* FJ930173 and *Favites* sp. EF089433) and sandy reef sediments (FJ 358860 and FJ358928), suggesting that closely related bacterial phylotypes can inhabit unrelated hosts and environments. Further studies are needed to determine whether these observations reflect a pattern of horizontal transmission of symbionts or transient taxa that were present at the time of sampling by chance.

Examining sponge-bacterial associations over larger spatio-temporal scales can be facilitated by microbial profiling techniques, such as DGGE and T-RFLP. Consistent with recent studies of sponge-bacteria symbioses [51], [62], T-RFLP recovered distinct microbial profiles and differentiated the unique bacterial communities present in sponges, tunicates and seawater. We documented consistent community-level trends despite the variable resolution of individual REs [75], indicating that this high-throughput and standardized technique will prove a useful tool in the study of sponge-bacteria associations.

Few studies have investigated the microbial communities associated with ascidians [76], [77] beyond the prominent cyanobacterial symbionts in the genera *Prochloron* and *Synechocystis* [78], [79]. To date, the most comprehensive analyses of microbial symbionts in ascidians have focused on a Mediterranean species, *Cystodytes dellechiajei*. This colonial ascidian was shown to host diverse bacterial and archaeal communities [80], [81] that may benefit the host ascidian directly by providing a food source (e.g., phagocytosis by host cells) or indirectly through the acquisition of nutrients (e.g., nitrification). Only 1 sequence derived from *Didemnum* sp. herein was closely related (97.8% identity) to previously reported clones from ascidians (*C. dellechiajei*), showing negligible symbiont community overlap between these hosts. Bacterial sequences from *Didemnum* sp. were more often closely related to sediment-derived and coral-associated clones. Clearly, additional studies of ascidian-associated microbes are required to understand the host-specificity and ecological roles of these symbionts; however, preliminary results show that ascidians host diverse bacterial symbionts related to other invertebrate-associated microbes, similar to findings from culture-based diversity studies [80], and suggest that ascidians should also be considered as potential niche habitats for specialized symbionts and hotspots of marine microbial diversity.

Early trends in the emerging field of sponge microbiology include the occurrence of sponge-specific bacteria that are distinct from bacterioplankton yet shared among diverse hosts from disparate geographic regions. In this study, the bacterial communities in *H. heliophila* and *H. tubifera* were shown to be markedly different, consisting of specialized symbionts distinct from the previously reported and widespread sponge-specific clusters. In *H. heliophila*, these symbionts were also present in conspecific host populations from the southwestern Atlantic, suggesting that specialist communities are maintained despite large geographic distances among host populations. In addition, the current study highlights the ability of T-RFLP analysis to produce rapid, accurate profiles of sponge-associated communities and thus its applicability to large-scale studies of spatio-temporal monitoring and experimentation. Future studies describing symbiont communities among diverse sponge hosts and targeting host-symbiont interactions will enhance our understanding of the selective pressures that shape these communities and further reveal the prevalence and trade-offs of hosting generalist versus specialist microbial communities. With implications ranging from basic sponge ecology and host-symbiont coevolution to natural products prospecting, the necessity and incentive for research in the field of sponge-microbial symbioses continues to increase.

## Materials and Methods

### Ethics Statement

No state or federal permits were required for these collections. In the State of Alabama, offshore drilling sites are owned by the State and are leased to various operators (often with rapid turnover) by the State. While the machinery and site are leased from the State, the waters surrounding the platforms remain accessible to the public. Platform operators do not regulate fishing or harvesting at the platforms; this right is retained by the State of Alabama. Thus, it is permissible to scuba dive, fish, and harvest organisms at each platform site; thus, these platforms have many recreational visitors every day. Legal permission to fish, harvest, or collect some types of organisms (e.g. fishes) must be obtained from the State of Alabama, but Alabama does not require permission to collect sponges. The United States federal government also does not require permission to collect sponges. Our fieldwork required permission from the Dauphin Island Sea Lab (DISL) to use their boat and crew to reach each field location. The DISL crew contacted each platform operator via radio for the logistical permission to approach the platform, which is necessary for safety reasons.

### Sample Collection and Species Identification

The marine sponges *Hymeniacidon heliophila*, *Haliclona tubifera* and *Halichondria* sp., the colonial tunicate *Didemnum* sp., and ambient seawater were collected from the pilings supporting 5 natural gas drilling platforms in the northern Gulf of Mexico (Table S9). Sponge and tunicate samples were processed individually and preserved in ethanol for morphological analyses and RNAlater (Ambion) for genetic analyses. Ambient seawater samples were collected directly next to sampled sponges in 500 mL Nalgene bottles, pre-filtered through a 55 µm mesh screen to remove debris and concentrated on 0.2 µm filters. Filters were immediately preserved in RNAlater for subsequent genetic analyses.

Sponge samples were identified by morphological analyses, using light microscopy of spicules and histological sections and the checklists and characters provided by Rützler et al. [82] and Little [83], and by molecular analyses, using a segment of nuclear ribosomal DNA corresponding to the 5′-end of the 28S subunit and the entire second internal transcribed spacer (ITS-2) region following the methods of Erwin & Thacker ([84]; GenBank accession numbers JF824781-JF824794). Species identifications were confirmed for *Hymeniacidon heliophila* and *Haliclona (Reniera) tubifera*, reported as *H. permollis* by Little [83]. *Halichondria* sp. was identified only to the genus level, as the two specimens collected did not match the morphology of any described species of *Halichondria* reported from the Gulf of Mexico [64], [82], but displayed the morphological characteristics of the genus and exhibited 94% sequence identity to a partial 28S rDNA sequence from *H. panicea* (GenBank accession number AF062607 [85]). *Halichondria* sp. colonies were rarely encountered on platform pilings, yielding only two collected specimens, and thus lacked proper replication for statistical comparisons. Therefore, bacterial sequences recovered from *Halichondria* sp. were only included in phylogenetic tree reconstructions for comparative analyses.

### Whole Genomic DNA Extractions

Metagenomic DNA extracts were prepared from sponge, tunicate, and concentrated seawater samples using the Wizard Genomic DNA Purification Kit (Promega) and cleaned using the Wizard DNA Clean-Up System (Promega). Prepared DNA extracts were used as templates in PCR amplification for both clone library construction and T-RFLP analyses.

### Clone Libraries and DNA Sequence Analysis

The universal bacterial forward primer Eco8F (5′-AGA GTT TGA TCA TGG CTC AG-3′) [86] and reverse primer 1509R (5′-GGT TAC CTT GTT ACG ACT T-3′) [87] were used in PCR reactions to amplify approximately 1,500 bp of the bacterial 16S rRNA gene sequence. Total PCR reaction volume was 50 µl, including 25 pmol of each primer, 10 nmol of each dNTP, 1X MasterTaq PCR Buffer (Eppendorf), and 1X TaqMaster additive (Eppendorf). Thermocycler reaction conditions for bacterial rRNA gene amplification were an initial denaturing time of 2 min at 94°C, followed by the addition of 0.5 units MasterTaq DNA polymerase (Eppendorf), then 34 cycles of 1 min at 94°C, 0.5 min at 50°C, and 1.5 min 72°C, and a final extension time of 2 min at 72°C. PCR products were gel-purified and cleaned using the Wizard SV Gel Clean-Up System (Promega) and ligated into plasmids using the pGEM T-Easy Vector System (Promega).

Individual clones were PCR-screened using vector primers until 15 clones with approximately 1,500 bp inserts were recovered from each sponge, tunicate and seawater sample. Plasmids from positive clones were harvested using the QIAprep Spin Miniprep Kit (Qiagen) and sequenced on an ABI 377 automated sequencer at the UAB Center for AIDS Research DNA Sequencing Core Facility. A single forward sequencing reaction was performed for all clones using a plasmid primer or the forward amplification primer. All sequences were trimmed to 600 bp starting at the highly conserved *E. coli* site 54, thereby excluding ambiguities on either end of the sequencing reaction, checked for chimeric origin using Bellerophon [88] and deposited in GenBank (accession numbers EU315321 to EU315680 and JF824738-JF824766; Table S1). Sequences were ascribed to operational taxonomic units (OTUs) by grouping in Sequencher (GeneCodes) according to 99% or greater sequence identity. Representative clones from common 99% OTUs were bi-directionally sequenced to retrieve near full-length 16S rRNA gene sequences (>1400 bp; GenBank accession numbers JF824767-JF824780) for phylogenetic analyses. Representative sequences from each 99% OTU were analyzed by using a nucleotide-nucleotide BLAST search [89] to find the most closely related sequence, and by using the Ribosomal Database Project II [90] sequence classifier to assess taxonomic affiliations. For each sequence, the highest percentage sequence identity match in GenBank was recorded, along with the major taxonomic group of this match. A likelihood ratio chi-square test was used to compare the frequency of major taxonomic groupings among sponge, tunicate, and seawater sources. *Alphaproteobacteria*, *Gammaproteobacteria*, and *Cyanobacteria* were analyzed as individual taxonomic groups, while *Bacteriodetes* and all other taxonomic groups were pooled due to a low frequency of occurrence. A log-linear model was used to compare the frequency of percentage sequence identities (grouped as: <95% identity, 95.0–96.9%, 97.0–98.9%, and 99–100%) that matched to GenBank sequences (grouped by isolation source as: sponge, seawater, sediment, coral, and other) among sponge, tunicate, and seawater sources.

Partial bacterial 16S rRNA gene sequences recovered herein, all *H. heliophila* derived clones from Turque et al. [32], and top BLAST search matches (total sequences = 734) were aligned using MAFFT [91], with an archaeal outgroup (*Haloarcula vallismortis*, GenBank accession number D50851 [92]). Maximum likelihood (ML) phylogenetic trees were constructed in RAxML [93] using the general time reversible (GTR) model of nucleotide substitutions, with a gamma distribution of substitution rate heterogeneity among sites; support for each node was assessed using 100 bootstrap replicates.

Recovered sponge and tunicate-associated bacteria were classified as either ‘specialist’ or ‘generalist’ symbionts based on host-specificity. Bacterial OTUs present only in sponge or tunicate samples and exhibiting ≥2% sequence divergence from free-living bacteria reported in GenBank were considered specialist symbionts. Bacterial OTUs shared with seawater communities and/or closely related (>98% identity) to environmental (i.e., non-symbiont) DNA sequences in GenBank were considered generalist symbionts. Specialist symbionts were further classified based on their abundance/presence in each community, with (1) ‘dominant symbionts’ present in all samples from one host species and accounting for over one-fourth of all recovered clones, (2) ‘common’ symbionts present in more than 1 sample from a host species and (3) ‘rare symbionts’ present in only 1 sample from a host species.

To compare the diversity of recovered bacterial communities, we calculated common ecological indices of diversity for each 15-clone sample: species richness (S_(obs)_), expected species richness (S_(Chao1)_), Shannon diversity index (H′), and Evenness (J). EstimateS software version 7.5 [94] was used to calculate the Chao1 expected richness and rarefaction curves for each 15-clone sample and for all samples within each source. Richness, evenness, and diversity of bacterial communities were compared across sponge, tunicate, and seawater sources using a one-way analysis of variance (ANOVA) with a Bonferroni correction applied to all pairwise post-hoc comparisons. Average rarefaction curves were compared among sources using an analysis of covariance (ANCOVA) of log-transformed data.

Bray-Curtis similarity matrices were constructed using square root transformations of relative OTU abundances and the presence/absence of OTUs in each sample and multi-dimensional scaling (MDS) plots were used to visually compare the bacterial communities recovered from each sample. Analysis of similarity (ANOSIM) was used to compare the statistical significance of similarity among bacterial communities recovered from sponge, tunicate, and seawater sources. Calculations were performed using the PRIMER v5.1.2 computer program (Plymouth Marine Laboratory, UK). Additionally, the LIBSHUFF program [95] was used to compare bacterial community similarity among sources. Both methods were used to assess community similarity because ANOSIM is a more conservative estimate, relying on OTU definitions, while LIBSHUFF is a more comprehensive estimate, incorporating all sequence information into the analysis.

Genetic diversity was compared among sponge, tunicate, and seawater sources using an analysis of molecular variance (AMOVA). Levels of variation included the source of the samples, replicates within each source, and sequences within replicates. Distances were calculated for AMOVA using the Tajima and Nei algorithm with alpha = 0.05. Using the Arlequin software package, version 3.0 [96], variation among sources was computed as F_ST_, with statistical significance based on 1000 permutations. Distributions of unique lineages among bacterial communities were examined using a phylogenetic lineage-sorting test (P-test) [97]. The net relatedness index (NRI) and nearest taxon index (NTI) were computed using PHYLOCOM [98], [99]; these metrics compare the phylogenetic dispersion and clustering of lineages within and among communities.

### T-RFLP Analysis

The universal bacterial forward primer Eco8F, tagged with a hexachlorofluorescein label (HEX), and reverse primer 1509R were used in PCR reactions to amplify approximately 1,500 bp of the bacterial 16S rRNA gene sequence. The total PCR reaction volume was 50 µl, including 15 pmol of the forward primer, 10 pmol of the reverse primer, 10 nmol of each dNTP, 1X MasterTaq PCR Buffer (Eppendorf), 1X TaqMaster additive (Eppendorf), and 2 units MasterTaq DNA Polymerase (Eppendorf). Thermocycler conditions consisted of an initial denaturing time of 5 min at 85°C, then 35 cycles of 0.75 min at 94°C, 1 min at 55°C, and 1.5 min at 72°C, with a final extension time of 10 min at 72°C. PCR products were gel-purified and cleaned using the Wizard SV Gel Clean-Up System (Promega). For each sample, PCR products from 3 separate PCR reactions were combined and quantified using a ND-1000 UV-Visible Spectrophotometer (NanoDrop®).

Approximately 400 ng of purified PCR products were digested with the restriction endonucleases *Hae*III, *Msp*I and *Rsa*I in a total reaction volume of 50 µl, following the manufacturer's protocol. All digests were incubated at 37°C for 8 hours. Immediately following digestion, samples were ethanol precipitated using 5 µl 3 M NaAc and 100 µl cold 100% ethanol. Samples were fully dried using a SpeedVac (LabConco).

Prior to capillary electrophoresis, 10 µl formamide and 0.5 µl GeneScan 500 TAMRA size standard were added to each sample. Samples were heated at 94°C for 2 min, immediately cooled on ice for 2 min, and analyzed on an automated sequencer (ABI377) with the program GeneScan (PE Applied Biosystems). Following electrophoresis, the length of individual fluorescently labeled terminal-restriction fragments (T-RFs) was determined by comparison with TAMRA size standards (Genescan™). Raw T-RFLP peak profiles were standardized using the variable threshold calculation across samples [100] and compared across samples using T-Align [101]. Peak profiles were standardized using relative abundance (percentage total fluorescence) and presence-absence (i.e., binary).

To compare the diversity of recovered bacterial communities, species richness (number of unique T-RFs per profile) was calculated for each sample and compared across source (sponges, tunicate, and seawater), restriction enzyme (*Hae*III, *Msp*I, and *Rsa*I) and the interaction of these two variables using a two-way analysis of variance (ANOVA). Bray-Curtis similarity matrices were constructed using square root transformations of relative T-RF abundances (percentage total fluorescence) and T-RF presence-absence in each sample and multi-dimensional scaling (MDS) plots were used to visually compare the bacterial communities recovered from each sample. Analysis of similarity (ANOSIM) was used to compare the statistical significance of similarity among bacterial communities recovered from sponge, tunicate, and seawater sources. Calculations were performed using the PRIMER v5.1.2 computer program (Plymouth Marine Laboratory, UK).

### Comparison of Clone Library and T-RFLP Analyses

A reference T-RF database (GOMB) was created using the 16S rRNA gene sequences recovered from clone libraries and used to compare predicted and empirical results from T-RFLP analysis, as well as, match individual T-RFs with 16S rRNA gene sequences. The GOMB database consisted of 5′-terminal fragment lengths, or reference T-RFs, for each 99% OTU (*n* = 139) for all restriction endonucleases (*n* = 3), as determined by *in silico* digestion using the computer software BioEdit [102]. The identities of empirical T-RFs were predicted by comparison to reference T-RFs and their corresponding gene sequences using the phylogenetic assignment tool (PAT, [103]). To account for discrepancies between predicted and empirical T-RFs (i.e., T-RF drift), which typically increase with T-RF size [104], bins were established with an increasing window of size tolerances to group all T-RFs within a given base pair range: T-RFs up to 200 bp in length received a tolerance bin of 1.0 bp, T-RFs from 201–400 bp in length received a tolerance bin of 1.5 bp, and T-RFs over 400 bp received a tolerance bin of 4.0 bp. PAT analyses were conducted individually for each of the three restriction enzymes used in T-RFLP analysis and composite profiles were constructed manually. For each 99% OTU (i.e., phylotype), a ‘T-RFLP signature’ was recovered, consisting of all empirically derived T-RFs that match predicted T-RFs using PAT analyses.

To assess potential phylogenetic biases of T-RFLP analysis (i.e., over- or under-represented bacterial taxa), the frequency of major taxonomic groups recovered by T-RFLP analyses were compared to the entire clone library using log-likelihood ratio goodness-of-fit (*G*) tests. *G*-tests were performed for each restriction enzyme, based on the observed frequency of major taxonomic groups among clone library phylotypes matched in T-RFLP profiles and the expected frequency of major taxonomic groups among all clone library phylotypes. To estimate the resolution of individual T-RFs in our dataset and the relatedness of microbial sequences sharing identical T-RFs, the incidence and sequence similarity of unique 16S rRNA gene sequences sharing the same empirically derived T-RF length in one or multiple restriction enzyme digests (i.e., T-RFLP signatures) was calculated.

## Supporting Information

Figure S1 **Phylogeny of bacterial 16S rRNA gene sequences recovered from sponges, tunicates and seawater.** Maximum likelihood phylogeny of 16S rRNA gene sequences recovered from sponges, tunicates and seawater with closely related GenBank sequences. Terminal nodes denote the host species or source of each sequence, followed by the GenBank accession number or sequence reference (HYM = *H. heliophila*, HTU = *Haliclona tubifera*, HCH = *Halichondria* sp., DID = *Didemnum* sp., GOM = Gulf of Mexico seawater). Numbers on nodes depict bootstrap support (100 replicates; values <50% not shown). Asterisks (**) indicate near full-length (>1400 bp) sequences.
(PDF)

Figure S2 **Average rarefaction curves for bacterial communities associated with sponge, tunicate and seawater samples.** Unique OTUs were encountered at a significantly faster rate in communities associated with a tunicate (*Didemnum* sp.) and seawater compared to the two sponge-associated bacterial communities (ANCOVA; *P*<0.05). Error bars represent ±1 SE.
(TIF)

Figure S3 **Average diversity (number of T-RFs) of bacterial communities associated with sponge, tunicate and ambient seawater samples.** Number of T-RFs per sample recovered from T-RFLP profiles using *Hae*III (black), *Msp*I (gray) and *Rsa*I (white). Asterisks denote significant differences (ANOVA; *P*<0.05) among enzymes. Error bars represent ±1 SE.
(TIF)

Figure S4 **Representative T-RFLP profiles (using restriction enzyme** ***Msp*** **I) of bacterial communities from sponges, tunicates and seawater.** Black peaks represent T-RFs within the accurate sizing range (100–500 bp). Vertical axis represents fluorescent units (note slight variation in scales) and horizontal axis T-RF length in base pairs. Isolation sources (left) are shown for each bacterial community profile.
(TIF)

Table S1 **Operational taxonomic unit (OTU), isolation source and GenBank accession numbers for all clones of bacterial 16S rRNA genes recovered from sponge (** ***Hymeniacidon heliophila*** **,** ***H. tubifera*** **and** ***Halichondria*** **sp.), tunicate (** ***Didemnum*** **sp.) and ambient seawater samples.**
(DOC)

Table S2 **Pairwise comparisons of sponge, tunicate and seawater bacterial community similarity (ANOSIM), highlighting the magnitude (R-statistic, top value) and significance (** ***P*** **-values, bottom value) of dissimilarity.**
(DOC)

Table S3 **Pairwise comparisons of the phylogenetic diversity (AMOVA, F_ST_) and phylogenetic relatedness (P-tests) of bacterial communities recovered from the sponge, tunicate and seawater samples.**
(DOC)

Table S4 **Net relatedness index (NRI) and nearest taxon index (NTI) of bacterial communities recovered from sponge, tunicate and seawater samples.**
(DOC)

Table S5 **Matches between empirically derived T-RFs and predicted T-RFs from clone library 16S rRNA gene sequences, using the restriction endonucleases** ***Hae*** **III,** ***Msp*** **I and** ***Rsa*** I. * = predicted T-RF size outside the sensitivity range of T-RFLP analysis (100–500 bp).
(DOC)

Table S6 **Individual T-RFs recovered using the enzyme** ***Hae*** **III and matching 16S rRNA gene sequence OTUs from clone library analyses.**
(DOC)

Table S7 **Individual T-RFs recovered using the enzyme** ***Msp*** **I and matching 16S rRNA gene sequence OTUs from clone library analyses.**
(DOC)

Table S8 **Individual T-RFs recovered using the enzyme** ***Rsa*** **I and matching 16S rRNA gene sequence OTUs from clone library analyses.**
(DOC)

Table S9 **Sample number, platform number and GPS coordinates of sponge, tunicate and seawater samples collected from drilling platform pilings in the northern Gulf of Mexico.**
(DOC)
