# Supplementary figures and images for: Phylogenetic Diversity, Host-Specificity and Community Profiling of Sponge-Associated Bacteria in the Northern Gulf of Mexico
Source: PLoS One. 2011 Nov 2;6(11):e26806. doi: 10.1371/journal.pone.0026806 (PMC3206846; doi:10.1371/journal.pone.0026806)

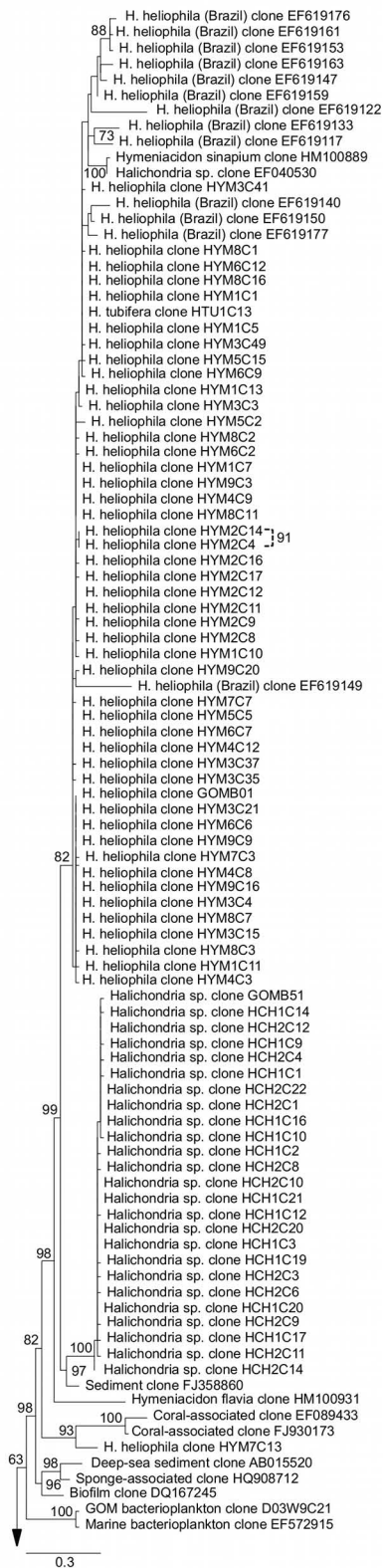

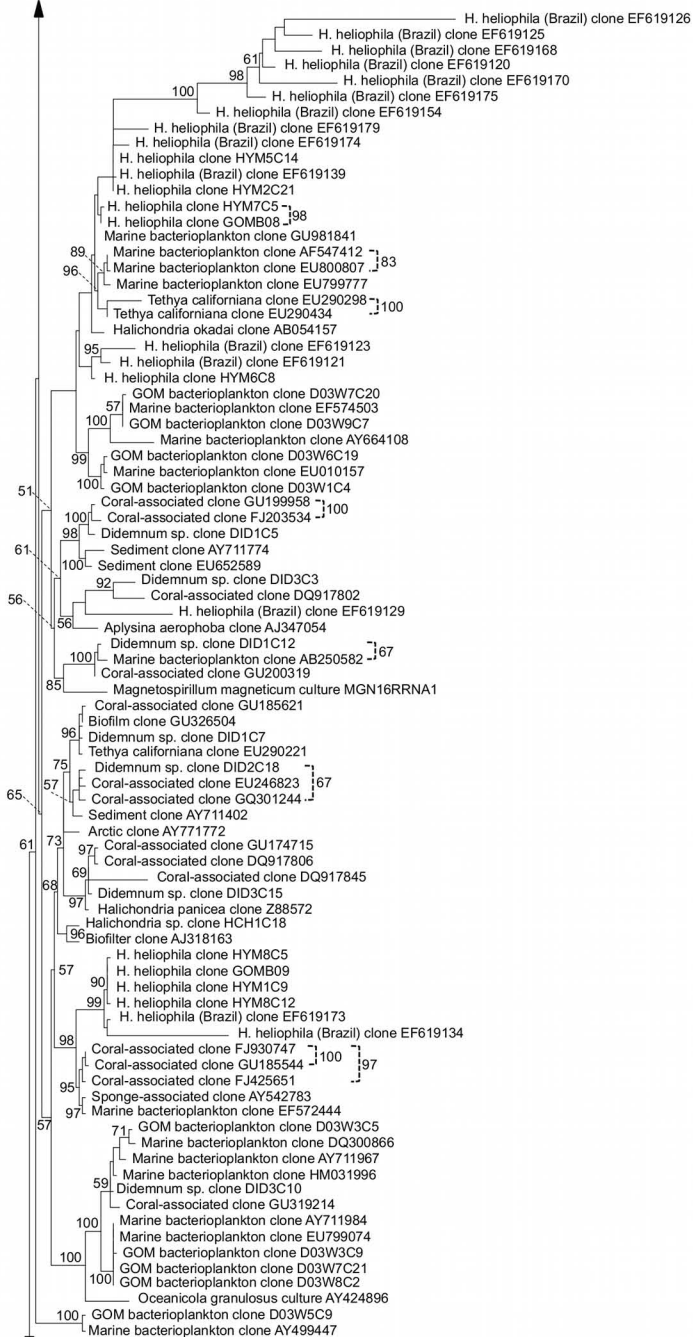

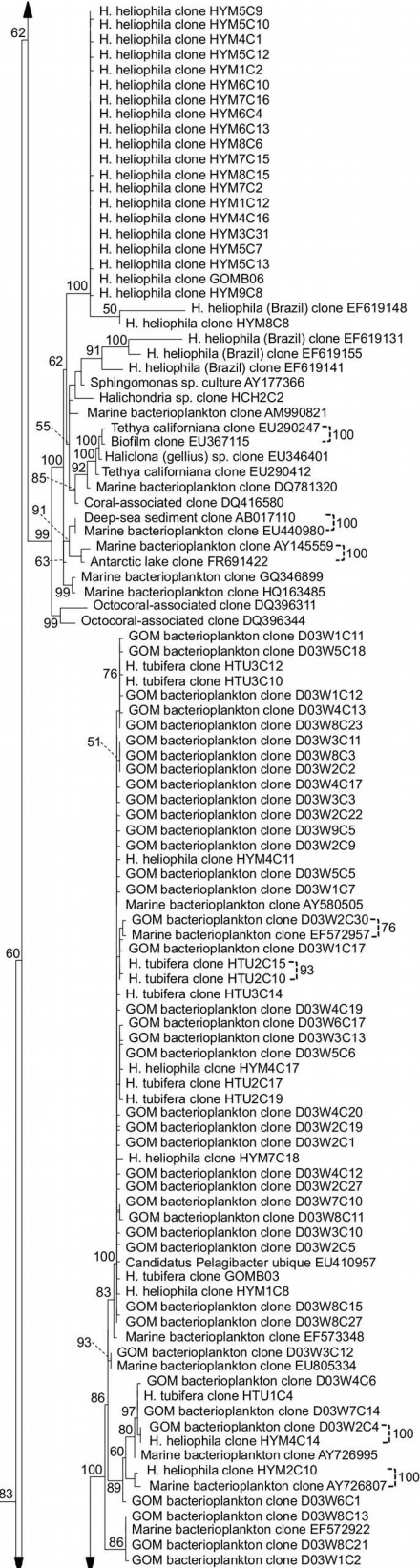

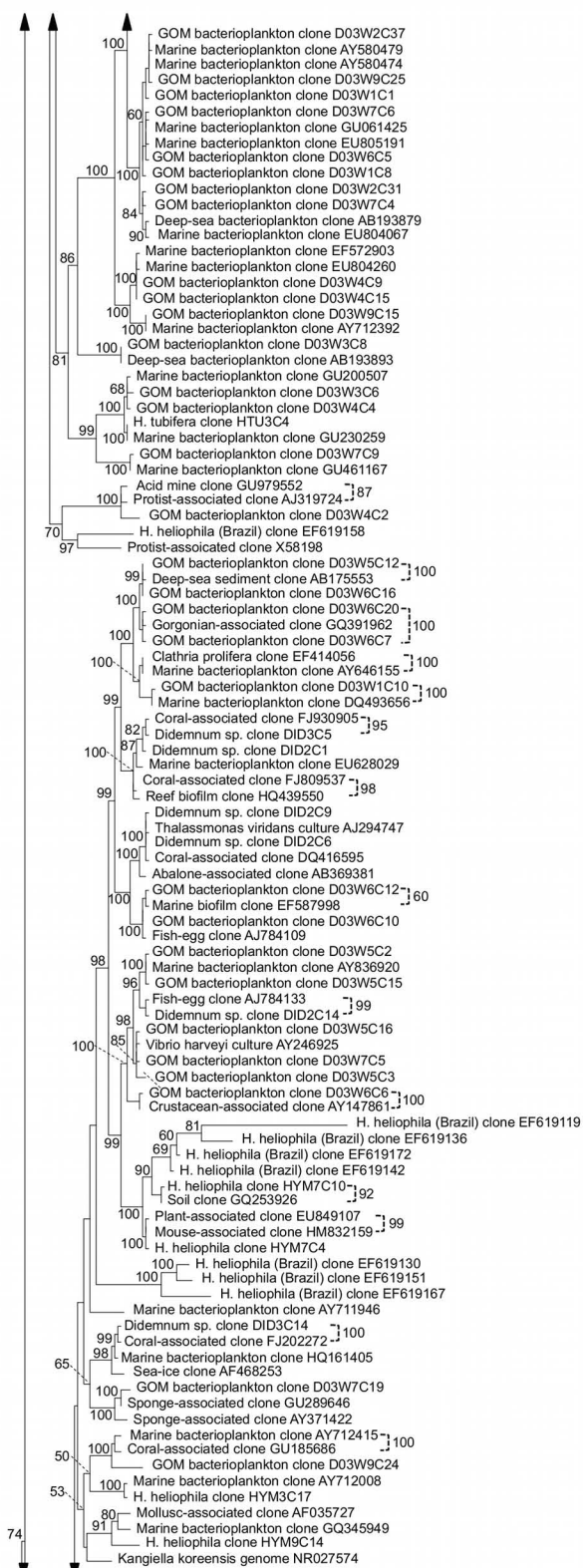

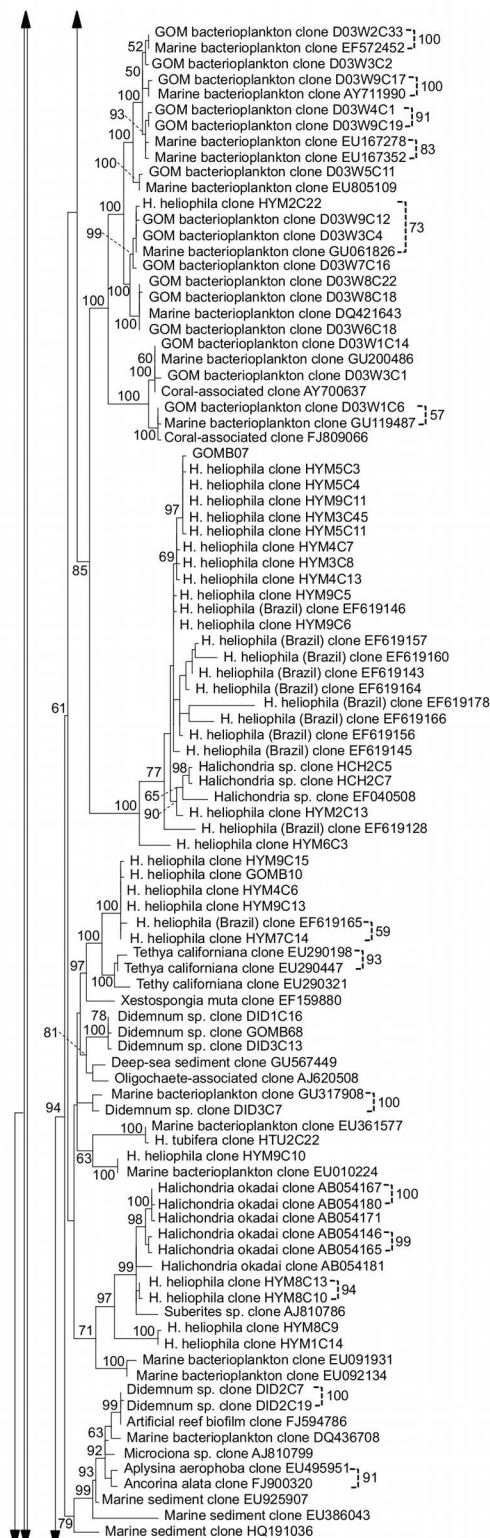

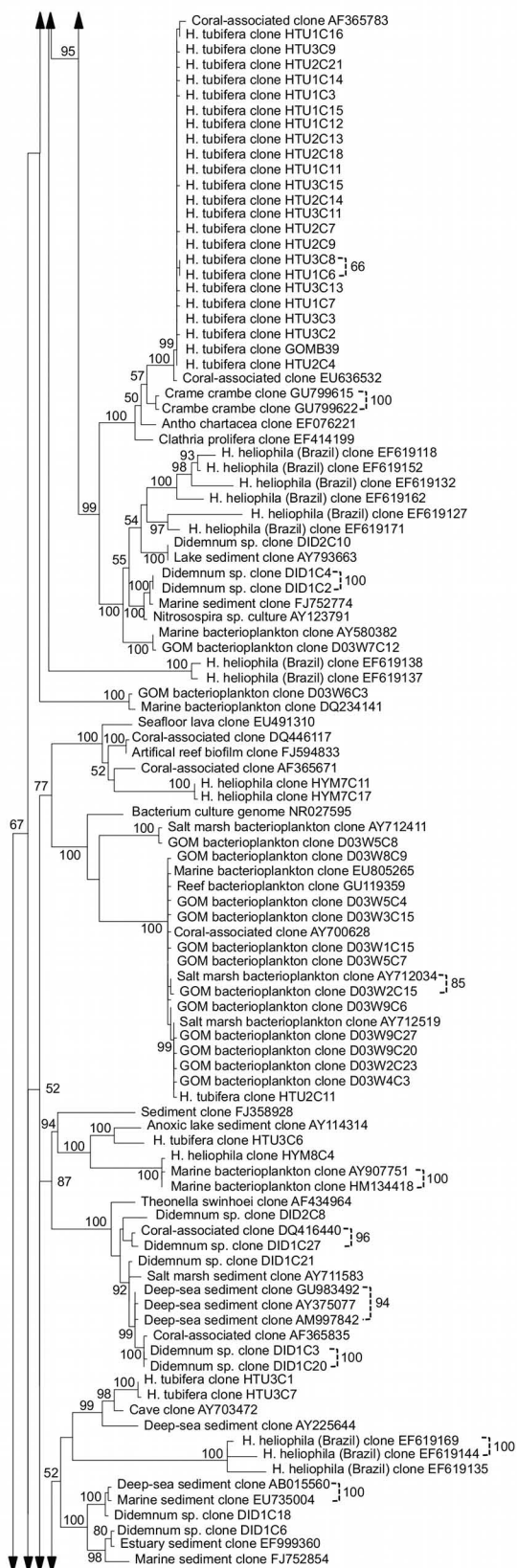

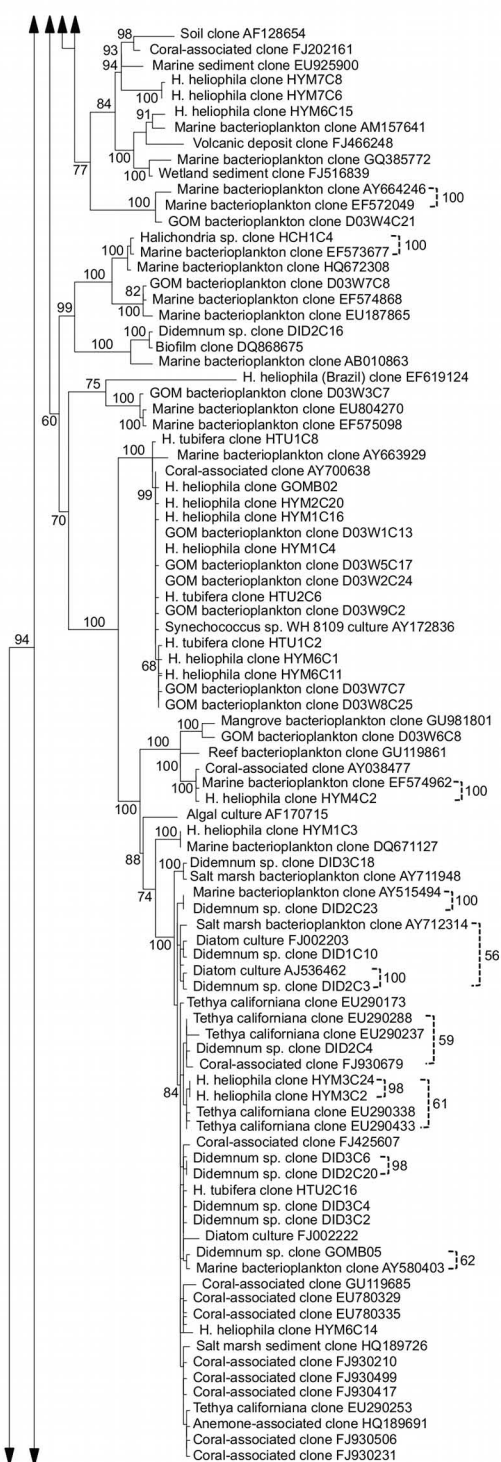

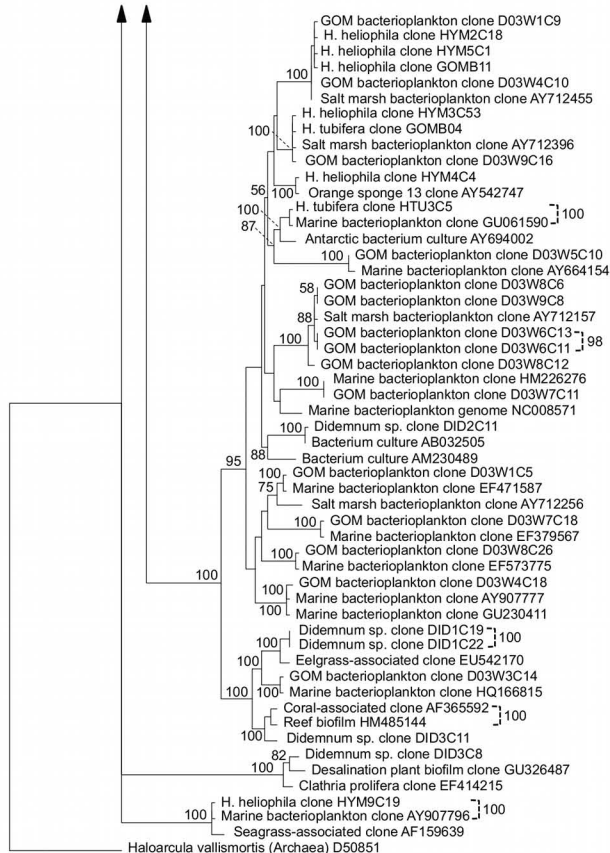

Supplement: Figure S1 — Phylogeny of bacterial 16S rRNA gene sequences recovered from sponges, tunicates and seawater. Maximum likelihood phylogeny of 16S rRNA gene sequences recovered from sponges, tunicates and seawater with closely related GenBank sequences. Terminal nodes denote the host species or source of each sequence, followed by the GenBank accession number or sequence reference (HYM = H. heliophila, HTU = Haliclona tubifera, HCH = Halichondria sp., DID = Didemnum sp., GOM = Gulf of Mexico seawater). Numbers on nodes depict bootstrap support (100 replicates; values <50% not shown). Asterisks (**) indicate near full-length (>1400 bp) sequences. (PDF) [file pone.0026806.s001.pdf]

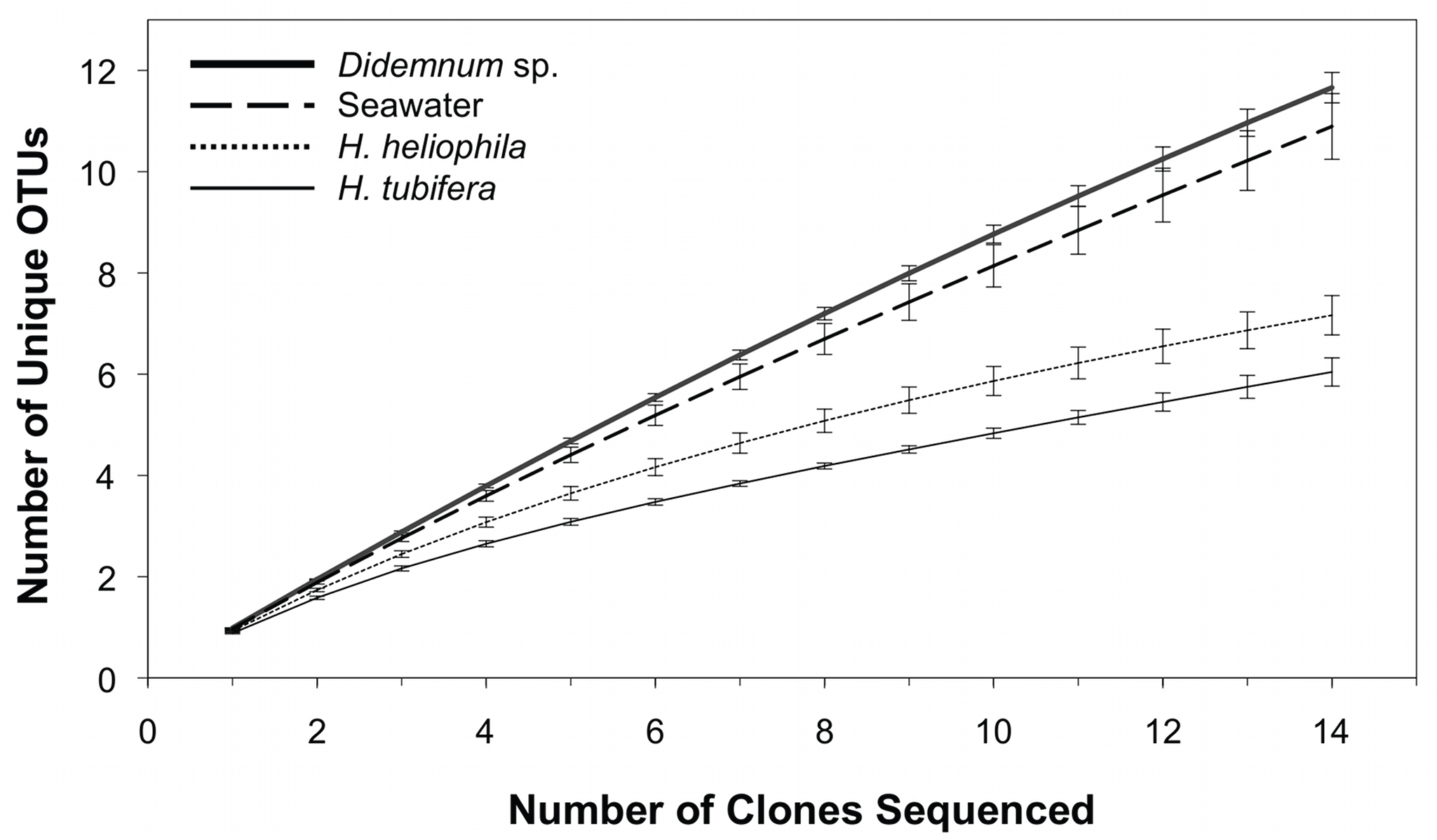

Supplement: Figure S2 — Average rarefaction curves for bacterial communities associated with sponge, tunicate and seawater samples. Unique OTUs were encountered at a significantly faster rate in communities associated with a tunicate (Didemnum sp.) and seawater compared to the two sponge-associated bacterial communities (ANCOVA; P<0.05). Error bars represent ±1 SE. (TIF) [file pone.0026806.s002.tif]

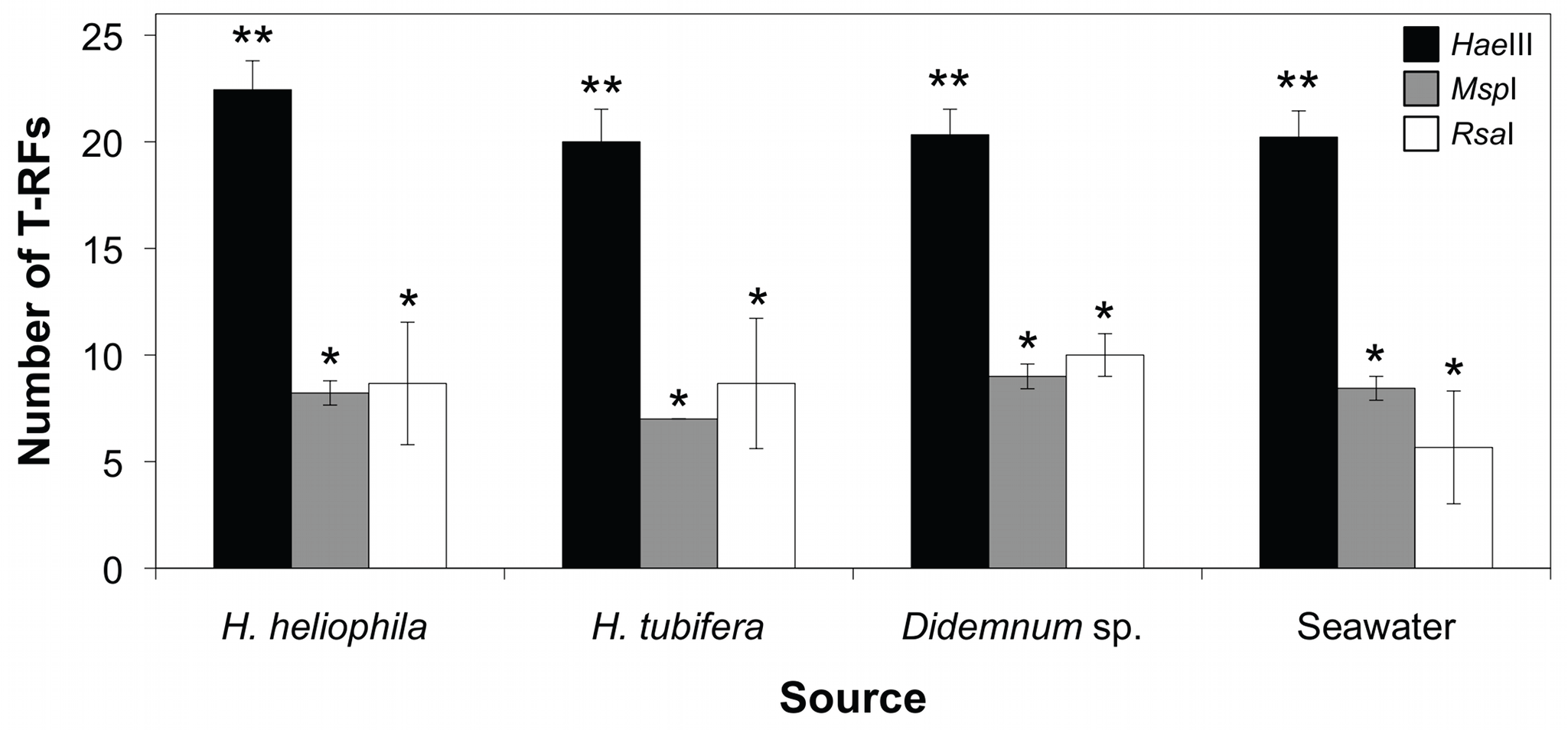

Supplement: Figure S3 — Average diversity (number of T-RFs) of bacterial communities associated with sponge, tunicate and ambient seawater samples. Number of T-RFs per sample recovered from T-RFLP profiles using HaeIII (black), MspI (gray) and RsaI (white). Asterisks denote significant differences (ANOVA; P<0.05) among enzymes. Error bars represent ±1 SE. (TIF) [file pone.0026806.s003.tif]

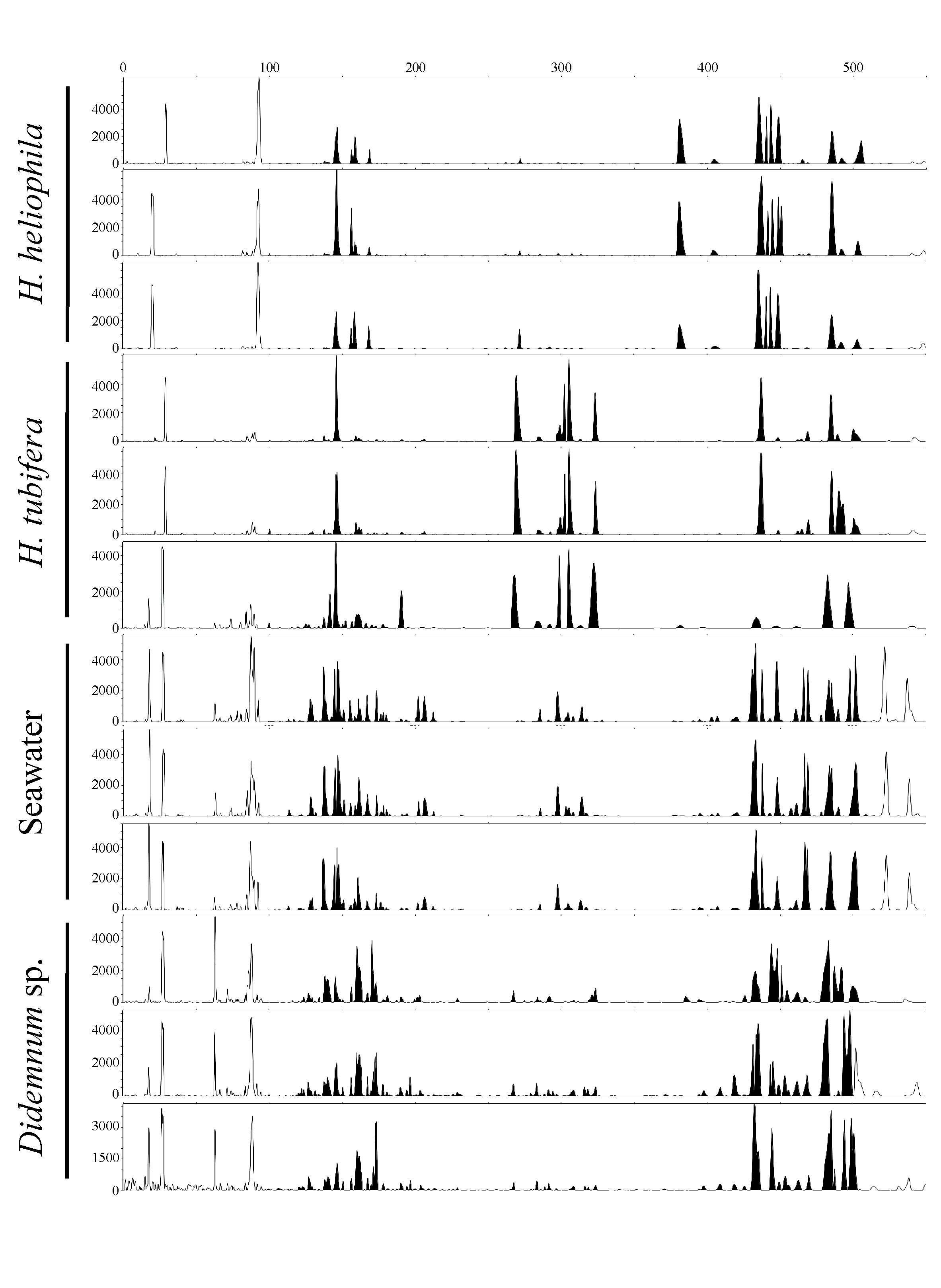

Supplement: Figure S4 — Representative T-RFLP profiles (using restriction enzyme Msp I) of bacterial communities from sponges, tunicates and seawater. Black peaks represent T-RFs within the accurate sizing range (100–500 bp). Vertical axis represents fluorescent units (note slight variation in scales) and horizontal axis T-RF length in base pairs. Isolation sources (left) are shown for each bacterial community profile. (TIF) [file pone.0026806.s004.tif]
