# Supplementary material for: Phylogenetic Diversity, Host-Specificity and Community Profiling of Sponge-Associated Bacteria in the Northern Gulf of Mexico
Source: PLoS One. 2011 Nov 2;6(11):e26806. doi: 10.1371/journal.pone.0026806 (PMC3206846; doi:10.1371/journal.pone.0026806)
Supplement: Table S1 — Operational taxonomic unit (OTU), isolation source and GenBank accession numbers for all clones of bacterial 16S rRNA genes recovered from sponge ( Hymeniacidon heliophila , H. tubifera and Halichondria sp.), tunicate ( Didemnum sp.) and ambient seawater samples. (DOC) [file pone.0026806.s005.doc]

**Table S1.** Operational taxonomic unit (OTU), isolation source and GenBank accession numbers for all clones of bacterial 16S rRNA genes recovered from sponge (*Hymeniacidon heliophila, H. tubifera* and *Halichondria* sp.), tunicate (*Didemnum* sp.) and ambient seawater samples.

| **99%**  **OTU** | **Isolation**  **Source** | **Individual**  **Clone ID** | **GenBank**  **Accession** | **99%**  **OTU** | **Isolation**  **Source** | **Individual Clone ID** | **GenBank**  **Accession** |
| --- | --- | --- | --- | --- | --- | --- | --- |
| GOMB1 | *H. heliophila* | HYM1-C1 | EU315321 | GOMB3 | *H. heliophila* | HYM1-C8 | EU315382 |
|  |  | HYM1-C5 | EU315322 |  |  | HYM4-C11 | EU315383 |
|  |  | HYM1-C7 | EU315323 |  |  | HYM4-C17 | EU315384 |
|  |  | HYM1-C10 | EU315324 |  | *H. tubifera* | HTU1-C10 | EU315385 |
|  |  | HYM1-C11 | EU315325 |  |  | HTU2-C10 | EU315386 |
|  |  | HYM1-C13 | EU315326 |  |  | HTU2-C15 | EU315387 |
|  |  | HYM2-C4 | EU315327 |  |  | HTU2-C17 | EU315388 |
|  |  | HYM2-C8 | EU315328 |  |  | HTU2-C19 | EU315389 |
|  |  | HYM2-C9 | EU315329 |  |  | HTU3-C10 | EU315390 |
|  |  | HYM2-C11 | EU315330 |  |  | HTU3-C12 | EU315391 |
|  |  | HYM2-C12 | EU315331 |  |  | HTU3-C14 | EU315392 |
|  |  | HYM2-C14 | EU315332 |  | Seawater | D03W1-C7 | EU315393 |
|  |  | HYM2-C16 | EU315333 |  |  | D03W1-C11 | EU315394 |
|  |  | HYM2-C17 | EU315334 |  |  | D03W1-C12 | EU315395 |
|  |  | HYM3-C3 | EU315335 |  |  | D03W1-C17 | EU315396 |
|  |  | HYM3-C4 | EU315336 |  |  | D03W2-C1 | EU315397 |
|  |  | HYM3-C15 | EU315337 |  |  | D03W2-C2 | EU315398 |
|  |  | HYM3-C21 | EU315338 |  |  | D03W2-C5 | EU315399 |
|  |  | HYM3-C35 | EU315339 |  |  | D03W2-C9 | EU315400 |
|  |  | HYM3-C37 | EU315340 |  |  | D03W2-C19 | EU315401 |
|  |  | HYM3-C41 | EU315341 |  |  | D03W2-C22 | EU315402 |
|  |  | HYM3-C49 | EU315342 |  |  | D03W2-C27 | EU315403 |
|  |  | HYM4-C3 | EU315343 |  |  | D03W3-C3 | EU315404 |
|  |  | HYM4-C8 | EU315344 |  |  | D03W3-C10 | EU315405 |
|  |  | HYM4-C9 | EU315345 |  |  | D03W3-C11 | EU315406 |
|  |  | HYM4-C12 | EU315346 |  |  | D03W3-C13 | EU315407 |
|  |  | HYM5-C5 | EU315347 |  |  | D03W4-C12 | EU315408 |
|  |  | HYM5-C6 | EU315348 |  |  | D03W4-C13 | EU315409 |
|  |  | HYM5-C15 | EU315349 |  |  | D03W4-C17 | EU315410 |
|  |  | HYM6-C2 | EU315350 |  |  | D03W4-C19 | EU315411 |
|  |  | HYM6-C6 | EU315351 |  |  | D03W4-C20 | EU315412 |
|  |  | HYM6-C7 | EU315352 |  |  | D03W5-C5 | EU315413 |
|  |  | HYM6-C9 | EU315353 |  |  | D03W5-C6 | EU315414 |
|  |  | HYM6-C12 | EU315354 |  |  | D03W5-C18 | EU315415 |
|  |  | HYM7-C3 | EU315355 |  |  | D03W6-C17 | EU315416 |
|  |  | HYM7-C7 | EU315356 |  |  | D03W7-C10 | EU315417 |
|  |  | HYM8-C1 | EU315357 |  |  | D03W8-C3 | EU315418 |
|  |  | HYM8-C2 | EU315358 |  |  | D03W8-C11 | EU315419 |
|  |  | HYM8-C3 | EU315359 |  |  | D03W8-C15 | EU315420 |
|  |  | HYM8-C7 | EU315360 |  |  | D03W8-C23 | EU315421 |
|  |  | HYM8-C11 | EU315361 |  |  | D03W8-C27 | EU315422 |
|  |  | HYM8-C16 | EU315362 |  |  | D03W9-C5 | EU315423 |
|  |  | HYM9-C3 | EU315363 | GOMB4 | *H. heliophila* | HYM3-C53 | EU315424 |
|  |  | HYM9-C9 | EU315364 |  | *H. tubifera* | HTU1-C9 | EU315425 |
|  |  | HYM9-C16 | EU315365 | GOMB5 | *H. tubifera* | HTU2-C16 | EU315426 |
|  |  | HYM9-C20 | EU315366 |  | *Didemnum* sp. | DID3-C2 | EU315427 |
|  | *H. tubifera* | HTU1-C13 | EU315367 |  |  | DID3-C4 | EU315428 |
| GOMB2 | *H. heliophila* | HYM1-C4 | EU315368 |  |  | DID3-C12 | EU315429 |
|  |  | HYM1-C6 | EU315369 | GOMB6 | *H. heliophila* | HYM1-C2 | EU315430 |
|  |  | HYM1-C16 | EU315370 |  |  | HYM1-C12 | EU315431 |
|  |  | HYM2-C20 | EU315371 |  |  | HYM3-C31 | EU315432 |
|  |  | HYM6-C1 | EU315372 |  |  | HYM4-C1 | EU315433 |
|  |  | HYM6-C11 | EU315373 |  |  | HYM4-C5 | EU315434 |
|  | *H. tubifera* | HTU1-C2 | EU315374 |  |  | HYM4-C16 | EU315435 |
|  |  | HTU2-C6 | EU315375 |  |  | HYM5-C7 | EU315436 |
|  | Seawater | D03W1-C13 | EU315376 |  |  | HYM5-C9 | EU315437 |
|  |  | D03W2-C24 | EU315377 |  |  | HYM5-C10 | EU315438 |
|  |  | D03W5-C17 | EU315378 |  |  | HYM5-C12 | EU315439 |
|  |  | D03W7-C7 | EU315379 |  |  | HYM5-C13 | EU315440 |
|  |  | D03W8-C25 | EU315380 | GOMB6 | *H. heliophila* | HYM6-C4 | EU315441 |
|  |  | D03W9-C2 | EU315381 |  |  | HYM6-C10 | EU315442 |

| **99%**  **OTU** | **Isolation**  **Source** | **Individual**  **Clone ID** | **GenBank**  **Accession** | **99%**  **OTU** | **Isolation**  **Source** | **Individual Clone ID** | **GenBank**  **Accession** |
| --- | --- | --- | --- | --- | --- | --- | --- |
| GOMB6 | *H. heliophila* | HYM6-C13 | EU315443 | GOMB30 | *H. heliophila* | HYM7-C11 | EU315504 |
|  |  | HYM7-C2 | EU315444 |  |  | HYM7-C17 | EU315505 |
|  |  | HYM7-C15 | EU315445 | GOMB31 | *H. heliophila* | HYM7-C13 | EU315506 |
|  |  | HYM7-C16 | EU315446 | GOMB32 | *H. heliophila* | HYM7-C18 | EU315507 |
|  |  | HYM8-C6 | EU315447 | GOMB33 | *H. heliophila* | HYM8-C4 | EU315508 |
|  |  | HYM8-C15 | EU315448 | GOMB34 | *H. heliophila* | HYM8-C8 | EU315509 |
|  |  | HYM9-C8 | EU315449 | GOMB35 | *H. heliophila* | HYM8-C10 | EU315510 |
| GOMB7 | *H. heliophila* | HYM3-C8 | EU315450 |  |  | HYM8-C13 | EU315511 |
|  |  | HYM3-C45 | EU315451 | GOMB36 | *H. heliophila* | HYM9-C10 | EU315512 |
|  |  | HYM4-C7 | EU315452 | GOMB37 | *H. heliophila* | HYM9-C14 | EU315513 |
|  |  | HYM4-C13 | EU315453 | GOMB38 | *H. heliophila* | HYM9-C19 | EU315514 |
|  |  | HYM5-C3 | EU315454 | GOMB39 | *H. tubifera* | HTU1-C1 | EU315515 |
|  |  | HYM5-C4 | EU315455 |  |  | HTU1-C3 | EU315516 |
|  |  | HYM5-C11 | EU315456 |  |  | HTU1-C6 | EU315517 |
|  |  | HYM6-C5 | EU315457 |  |  | HTU1-C7 | EU315518 |
|  |  | HYM9-C5 | EU315458 |  |  | HTU1-C11 | EU315519 |
|  |  | HYM9-C6 | EU315459 |  |  | HTU1-C12 | EU315520 |
|  |  | HYM9-C11 | EU315460 |  |  | HTU1-C14 | EU315521 |
| GOMB8 | *H. heliophila* | HYM2-C21 | EU315461 |  |  | HTU1-C15 | EU315522 |
|  |  | HYM5-C8 | EU315462 |  |  | HTU1-C16 | EU315523 |
|  |  | HYM5-C14 | EU315463 |  |  | HTU2-C4 | EU315524 |
|  |  | HYM7-C5 | EU315464 |  |  | HTU2-C7 | EU315525 |
| GOMB9 | *H. heliophila* | HYM1-C9 | EU315465 |  |  | HTU2-C9 | EU315526 |
|  |  | HYM8-C5 | EU315466 |  |  | HTU2-C13 | EU315527 |
|  |  | HYM8-C12 | EU315467 |  |  | HTU2-C14 | EU315528 |
|  |  | HYM9-C7 | EU315468 |  |  | HTU2-C18 | EU315529 |
| GOMB10 | *H. heliophila* | HYM4-C6 | EU315469 |  |  | HTU2-C21 | EU315530 |
|  |  | HYM7-C14 | EU315470 |  |  | HTU3-C2 | EU315531 |
|  |  | HYM9-C1 | EU315471 |  |  | HTU3-C3 | EU315532 |
|  |  | HYM9-C13 | EU315472 |  |  | HTU3-C8 | EU315533 |
|  |  | HYM9-C15 | EU315473 |  |  | HTU3-C9 | EU315534 |
| GOMB11 | *H. heliophila* | HYM2-C7 | EU315474 |  |  | HTU3-C11 | EU315535 |
|  |  | HYM2-C18 | EU315475 |  |  | HTU3-C13 | EU315536 |
|  |  | HYM5-C1 | EU315476 |  |  | HTU3-C15 | EU315537 |
|  | Seawater | D03W1-C9 | EU315477 | GOMB41 | *H. tubifera* | HTU1-C4 | EU315538 |
|  |  | D03W4-C10 | EU315478 |  | Seawater | D03W4-C6 | EU315539 |
| GOMB12 | *H. heliophila* | HYM1-C14 | EU315479 |  |  | D03W7-C14 | EU315540 |
|  |  | HYM8-C9 | EU315480 | GOMB42 | *H. tubifera* | HTU1-C8 | EU315541 |
| GOMB13 | *H. heliophila* | HYM1-C3 | EU315481 | GOMB43 | *H. tubifera* | HTU2-C11 | EU315542 |
| GOMB14 | *H. heliophila* | HYM2-C10 | EU315482 |  | Seawater | D03W2-C23 | EU315543 |
| GOMB15 | *H. heliophila* | HYM2-C13 | EU315483 |  |  | D03W4-C3 | EU315544 |
| GOMB16 | *H. heliophila* | HYM2-C22 | EU315484 |  |  | D03W9-C20 | EU315545 |
|  | Seawater | D03W3-C4 | EU315485 |  |  | D03W9-C27 | EU315546 |
|  |  | D03W7-C16 | EU315486 | GOMB44 | *H. tubifera* | HTU2-C22 | EU315547 |
|  |  | D03W9-C12 | EU315487 | GOMB47 | *H. tubifera* | HTU3-C1 | EU315548 |
| GOMB17 | *H. heliophila* | HYM3-C2 | EU315488 |  |  | HTU3-C7 | EU315549 |
|  |  | HYM3-C24 | EU315489 | GOMB48 | *H. tubifera* | HTU3-C4 | EU315550 |
| GOMB18 | *H. heliophila* | HYM3-C17 | EU315490 | GOMB49 | *H. tubifera* | HTU3-C5 | EU315551 |
| GOMB19 | *H. heliophila* | HYM4-C2 | EU315491 | GOMB50 | *H. tubifera* | HTU3-C6 | EU315552 |
| GOMB20 | *H. heliophila* | HYM4-C4 | EU315492 |  |  |  |  |
| GOMB21 | *H. heliophila* | HYM4-C14 | EU315493 | GOMB51 | *Halichondria* sp. | HCH1-C1 | JF824738 |
|  | Seawater | D03W2-C4 | EU315494 |  |  | HCH1-C2 | JF824746 |
| GOMB22 | *H. heliophila* | HYM5-C2 | EU315495 |  |  | HCH1-C3 | JF824749 |
| GOMB23 | *H. heliophila* | HYM6-C3 | EU315496 |  |  | HCH1-C9 | JF824751 |
| GOMB24 | *H. heliophila* | HYM6-C8 | EU315497 |  |  | HCH1-C10 | JF824739 |
| GOMB25 | *H. heliophila* | HYM6-C14 | EU315498 |  |  | HCH1-C12 | JF824740 |
| GOMB26 | *H. heliophila* | HYM6-C15 | EU315499 |  |  | HCH1-C14 | JF824741 |
| GOMB27 | *H. heliophila* | HYM7-C4 | EU315500 |  |  | HCH1-C16 | JF824742 |
| GOMB28 | *H. heliophila* | HYM7-C6 | EU315501 |  |  | HCH1-C17 | JF824743 |
|  |  | HYM7-C8 | EU315502 |  |  | HCH1-C19 | JF824745 |
| GOMB29 | *H. heliophila* | HYM7-C10 | EU315503 |  |  | HCH1-C20 | JF824747 |

| **99%**  **OTU** | **Isolation**  **Source** | **Individual**  **Clone ID** | **GenBank**  **Accession** | **99%**  **OTU** | **Isolation**  **Source** | **Individual Clone ID** | **GenBank**  **Accession** |
| --- | --- | --- | --- | --- | --- | --- | --- |
| GOMB51 | *Halichondria* sp. | HCH1-C21 | JF824748 | GOMB102 | Seawater | D03W2-C15 | EU315596 |
|  |  | HCH2-C1 | JF824752 |  |  | D03W3-C15 | EU315597 |
|  |  | HCH2-C3 | JF824760 |  |  | D03W5-C4 | EU315598 |
|  |  | HCH2-C4 | JF824761 |  |  | D03W5-C7 | EU315599 |
|  |  | HCH2-C6 | JF824763 |  |  | D03W8-C9 | EU315600 |
|  |  | HCH2-C8 | JF824765 |  |  | D03W9-C6 | EU315601 |
|  |  | HCH2-C9 | JF824766 | GOMB103 | Seawater | D03W1-C1 | EU315602 |
|  |  | HCH2-C10 | JF824753 |  |  | D03W1-C8 | EU315603 |
|  |  | HCH2-C11 | JF824754 |  |  | D03W6-C5 | EU315604 |
|  |  | HCH2-C12 | JF824755 |  |  | D03W7-C6 | EU315605 |
|  |  | HCH2-C14 | JF824756 |  |  | D03W9-C25 | EU315606 |
|  |  | HCH2-C20 | JF824758 | GOMB104 | Seawater | D03W6-C11 | EU315607 |
|  |  | HCH2-C22 | JF824759 |  |  | D03W6-C13 | EU315608 |
| GOMB52 | *Halichondria* sp. | HCH1-C4 | JF824750 |  |  | D03W8-C6 | EU315609 |
| GOMB53 | *Halichondria* sp. | HCH1-C18 | JF824744 |  |  | D03W9-C8 | EU315610 |
| GOMB54 | *Halichondria* sp. | HCH2-C2 | JF824757 | GOMB105 | Seawater | D03W1-C2 | EU315611 |
| GOMB55 | *Halichondria* sp. | HCH2-C5 | JF824762 |  |  | D03W3-C12 | EU315612 |
|  |  | HCH2-C7 | JF824764 |  |  | D03W8-C13 | EU315613 |
| GOMB68 | *Didemnum* sp. | DID1-C16 | EU315553 |  |  | D03W8-C21 | EU315614 |
|  |  | DID3-C9 | EU315554 | GOMB106 | Seawater | D03W3-C9 | EU315615 |
|  |  | DID3-C13 | EU315555 |  |  | D03W7-C21 | EU315616 |
| GOMB69 | *Didemnum* sp. | DID2-C20 | EU315556 |  |  | D03W8-C2 | EU315617 |
|  |  | DID3-C6 | EU315557 | GOMB107 | Seawater | D03W6-C18 | EU315618 |
| GOMB70 | *Didemnum* sp. | DID1-C2 | EU315558 |  |  | D03W8-C18 | EU315619 |
|  |  | DID1-C4 | EU315559 |  |  | D03W8-C22 | EU315620 |
| GOMB71 | *Didemnum* sp. | DID1-C3 | EU315560 | GOMB108 | Seawater | D03W4-C9 | EU315621 |
| GOMB71 | *Didemnum* sp. | DID1-C20 | EU315561 |  |  | D03W4-C15 | EU315622 |
| GOMB72 | *Didemnum* sp. | DID1-C5 | EU315562 | GOMB109 | Seawater | D03W4-C1 | EU315623 |
| GOMB73 | *Didemnum* sp. | DID1-C6 | EU315563 |  |  | D03W9-C19 | EU315624 |
| GOMB74 | *Didemnum* sp. | DID1-C7 | EU315564 | GOMB110 | Seawater | D03W5-C12 | EU315625 |
| GOMB75 | *Didemnum* sp. | DID1-C10 | EU315565 |  |  | D03W6-C16 | EU315626 |
| GOMB76 | *Didemnum* sp. | DID1-C12 | EU315566 | GOMB111 | Seawater | D03W5-C16 | EU315627 |
| GOMB77 | *Didemnum* sp. | DID1-C18 | EU315567 |  |  | D03W7-C5 | EU315628 |
| GOMB78 | *Didemnum* sp. | DID1-C19 | EU315568 | GOMB112 | Seawater | D03W1-C4 | EU315629 |
|  |  | DID1-C22 | EU315569 |  |  | D03W6-C19 | EU315630 |
| GOMB79 | *Didemnum* sp. | DID1-C21 | EU315570 | GOMB113 | Seawater | D03W7-C20 | EU315631 |
| GOMB80 | *Didemnum* sp. | DID1-C27 | EU315571 |  |  | D03W9-C7 | EU315632 |
| GOMB81 | *Didemnum* sp. | DID2-C1 | EU315572 | GOMB114 | Seawater | D03W5-C2 | EU315633 |
| GOMB82 | *Didemnum* sp. | DID2-C3 | EU315573 |  |  | D03W5-C15 | EU315634 |
| GOMB83 | *Didemnum* sp. | DID2-C4 | EU315574 | GOMB115 | Seawater | D03W6-C10 | EU315635 |
| GOMB84 | *Didemnum* sp. | DID2-C6 | EU315575 |  |  | D03W6-C12 | EU315636 |
|  |  | DID2-C9 | EU315576 | GOMB116 | Seawater | D03W1-C5 | EU315637 |
| GOMB85 | *Didemnum* sp. | DID2-C7 | EU315577 | GOMB117 | Seawater | D03W1-C6 | EU315638 |
|  |  | DID2-C19 | EU315578 | GOMB118 | Seawater | D03W1-C10 | EU315639 |
| GOMB86 | *Didemnum* sp. | DID2-C8 | EU315579 | GOMB119 | Seawater | D03W1-C14 | EU315640 |
| GOMB87 | *Didemnum* sp. | DID2-C10 | EU315580 | GOMB120 | Seawater | D03W2-C30 | EU315641 |
| GOMB88 | *Didemnum* sp. | DID2-C11 | EU315581 | GOMB121 | Seawater | D03W2-C31 | EU315642 |
| GOMB89 | *Didemnum* sp. | DID2-C14 | EU315582 | GOMB122 | Seawater | D03W2-C33 | EU315643 |
| GOMB90 | *Didemnum* sp. | DID2-C16 | EU315583 | GOMB123 | Seawater | D03W2-C37 | EU315644 |
| GOMB91 | *Didemnum* sp. | DID2-C18 | EU315584 | GOMB124 | Seawater | D03W3-C1 | EU315645 |
| GOMB92 | *Didemnum* sp. | DID2-C23 | EU315585 | GOMB125 | Seawater | D03W3-C2 | EU315646 |
| GOMB93 | *Didemnum* sp. | DID3-C3 | EU315586 | GOMB126 | Seawater | D03W3-C5 | EU315647 |
| GOMB94 | *Didemnum* sp. | DID3-C5 | EU315587 | GOMB127 | Seawater | D03W3-C6 | EU315648 |
| GOMB95 | *Didemnum* sp. | DID3-C7 | EU315588 | GOMB128 | Seawater | D03W3-C7 | EU315649 |
| GOMB96 | *Didemnum* sp. | DID3-C8 | EU315589 | GOMB129 | Seawater | D03W3-C8 | EU315650 |
| GOMB97 | *Didemnum* sp. | DID3-C10 | EU315590 | GOMB130 | Seawater | D03W3-C14 | EU315651 |
| GOMB98 | *Didemnum* sp. | DID3-C11 | EU315591 | GOMB131 | Seawater | D03W4-C2 | EU315652 |
| GOMB99 | *Didemnum* sp. | DID3-C14 | EU315592 | GOMB132 | Seawater | D03W4-C4 | EU315653 |
| GOMB100 | *Didemnum* sp. | DID3-C15 | EU315593 | GOMB133 | Seawater | D03W4-C18 | EU315654 |
| GOMB101 | *Didemnum* sp. | DID3-C18 | EU315594 | GOMB134 | Seawater | D03W4-C21 | EU315655 |
| GOMB102 | Seawater | D03W1-C15 | EU315595 | GOMB135 | Seawater | D03W5-C3 | EU315656 |

| **99%**  **OTU** | **Isolation**  **Source** | **Individual**  **Clone ID** | **GenBank**  **Accession** | **99%**  **OTU** | **Isolation**  **Source** | **Individual Clone ID** | **GenBank**  **Accession** |
| --- | --- | --- | --- | --- | --- | --- | --- |
| GOMB136 | Seawater | D03W5-C8 | EU315657 | GOMB148 | Seawater | D03W7-C9 | EU315669 |
| GOMB137 | Seawater | D03W5-C9 | EU315658 | GOMB149 | Seawater | D03W7-C11 | EU315670 |
| GOMB138 | Seawater | D03W5-C10 | EU315659 | GOMB150 | Seawater | D03W7-C12 | EU315671 |
| GOMB139 | Seawater | D03W5-C11 | EU315660 | GOMB151 | Seawater | D03W7-C18 | EU315672 |
| GOMB140 | Seawater | D03W6-C1 | EU315661 | GOMB152 | Seawater | D03W7-C19 | EU315673 |
| GOMB141 | Seawater | D03W6-C3 | EU315662 | GOMB153 | Seawater | D03W8-C12 | EU315674 |
| GOMB142 | Seawater | D03W6-C6 | EU315663 | GOMB154 | Seawater | D03W8-C26 | EU315675 |
| GOMB143 | Seawater | D03W6-C7 | EU315664 | GOMB155 | Seawater | D03W9-C15 | EU315676 |
| GOMB144 | Seawater | D03W6-C8 | EU315665 | GOMB156 | Seawater | D03W9-C16 | EU315677 |
| GOMB145 | Seawater | D03W6-C20 | EU315666 | GOMB157 | Seawater | D03W9-C17 | EU315678 |
| GOMB146 | Seawater | D03W7-C4 | EU315667 | GOMB158 | Seawater | D03W9-C21 | EU315679 |
| GOMB147 | Seawater | D03W7-C8 | EU315668 | GOMB159 | Seawater | D03W9-C24 | EU315680 |
