# Supplementary material for: Phylogenetic Diversity, Host-Specificity and Community Profiling of Sponge-Associated Bacteria in the Northern Gulf of Mexico
Source: PLoS One. 2011 Nov 2;6(11):e26806. doi: 10.1371/journal.pone.0026806 (PMC3206846; doi:10.1371/journal.pone.0026806)
Supplement: Table S2 — Pairwise comparisons of sponge, tunicate and seawater bacterial community similarity (ANOSIM), highlighting the magnitude (R-statistic, top value) and significance ( P -values, bottom value) of dissimilarity. (DOC) [file pone.0026806.s006.doc]

**Table S2.** Pairwise comparisons of sponge, tunicate and seawater bacterial community similarity (ANOSIM), highlighting the magnitude (R-statistic, top value) and significance (*P*-values, bottom value) of dissimilarity.

| Pairwise Comparison | Clones  (RA) | Clones  (P-A) | *Hae*III  (RA) | *Hae*III  (P-A) | *Msp*I  (RA) | *Msp*I  (P-A) | *Rsa*I  (RA) | *Rsa*I  (P-A) |
| --- | --- | --- | --- | --- | --- | --- | --- | --- |
| *H. heliophila* vs. *H. tubifera* | 0.962  ** | 0.776  ** | 1.000  ** | 1.000  ** | 0.728  * | 0.773  ** | 0.835  ** | 0.831  ** |
| *H. heliophila* vs. *Didemnum* sp. | 0.974  ** | 0.974  ** | 0.985  ** | 0.985  ** | 0.903  ** | 0.906  ** | 0.763  ** | 0.789  ** |
| *H. heliophila* vs. Seawater | 0.942  *** | 0.888  *** | 0.995  *** | 0.995  *** | 0.775  *** | 0.781  *** | 0.736  *** | 0.789  *** |
| *H. tubifera*. vs. *Didemnum* sp. | 0.741  (*P*=0.10) | 0.741  (*P*=0.10) | 0.963  (*P*=0.10) | 0.963  (*P*=0.10) | 1.000  (*P*=0.10) | 1.000  (*P*=0.10) | 0.667  (*P*=0.10) | 0.667  (*P*=0.10) |
| *H. tubifera*. vs. Seawater | 0.392  * | 0.311  (*P*=0.06) | 1.000  ** | 1.000  ** | 0.778  ** | 0.756  ** | 0.345  (*P*=0.06) | 0.375  * |
| *Didemnum* sp. vs. Seawater | 0.974  ** | 0.974  ** | 1.000  ** | 1.000  ** | 0.677  ** | 0.660  ** | 0.643  ** | 0.706  ** |

* = *P* ≤ 0.05, ** = *P* ≤ 0.005, *** = *P* ≤ 0.001

Pairwise analyses of similarity (ANOSIMs) were conducted using relative abundance data (RA) and presence-absence data (P-A) from clone library analyses (Clones) and T-RFLP analyses with 3 restriction endonucleases (*Hae*III, *Msp*I and *Rsa*I).
