# Supplementary material for: Phylogenetic Diversity, Host-Specificity and Community Profiling of Sponge-Associated Bacteria in the Northern Gulf of Mexico
Source: PLoS One. 2011 Nov 2;6(11):e26806. doi: 10.1371/journal.pone.0026806 (PMC3206846; doi:10.1371/journal.pone.0026806)
Supplement: Table S3 — Pairwise comparisons of the phylogenetic diversity (AMOVA, FST) and phylogenetic relatedness (P-tests) of bacterial communities recovered from the sponge, tunicate and seawater samples. (DOC) [file pone.0026806.s007.doc]

**Table S3.** Pairwise comparisons of the phylogenetic diversity (AMOVA, FST) and phylogenetic relatedness (P-tests) of bacterial communities recovered from the sponge, tunicate and seawater samples.

| Pairwise Comparison | F_ST_ | *P* | *P*-test |
| --- | --- | --- | --- |
| *H. tubifera* sp. vs. *H. heliophila* | 0.21260 | < 0.001* | 0.815 |
| *H. tubifera* vs. Seawater | 0.15646 | < 0.001* | 0.620 |
| *H. tubifera* vs. *Didemnum* sp. | 0.13920 | < 0.001* | 0.235 |
| *H. heliophila* vs. Seawater | 0.11424 | < 0.001* | < 0.001* |
| *H. heliophila* vs. *Didemnum* sp. | 0.11961 | < 0.001* | 0.005* |
| Seawater vs. *Didemnum* sp. | 0.10244 | < 0.001* | < 0.001* |

*F_ST_ *P* values and *P*-test values less than 0.05 indicate significantly dissimilar bacterial communities.
