# Supplementary material for: Phylogenetic Diversity, Host-Specificity and Community Profiling of Sponge-Associated Bacteria in the Northern Gulf of Mexico
Source: PLoS One. 2011 Nov 2;6(11):e26806. doi: 10.1371/journal.pone.0026806 (PMC3206846; doi:10.1371/journal.pone.0026806)
Supplement: Table S4 — Net relatedness index (NRI) and nearest taxon index (NTI) of bacterial communities recovered from sponge, tunicate and seawater samples. (DOC) [file pone.0026806.s008.doc]

**Table S4.** Net relatedness index (NRI) and nearest taxon index (NTI) of bacterial communities recovered from sponge, tunicate and seawater samples.

| Source | No. Taxa | NRI | *P* | NTI | *P* |
| --- | --- | --- | --- | --- | --- |
| *H. heliophila* | 135 | 6.700097 | <0.001* | 4.080797 | <0.001* |
| *H. tubifera* | 45 | 3.445091 | <0.001* | 2.205370 | 0.010* |
| Seawater | 135 | 0.945717 | 0.179 | 1.158574 | 0.138 |
| *Didemnum* sp. | 45 | -1.332680 | 0.901 | 0.413722 | 0.352 |

## **P* values less than 0.05 indicate phylogenetically under-dispersed communities.
