# Supplementary material for: Phylogenetic Diversity, Host-Specificity and Community Profiling of Sponge-Associated Bacteria in the Northern Gulf of Mexico
Source: PLoS One. 2011 Nov 2;6(11):e26806. doi: 10.1371/journal.pone.0026806 (PMC3206846; doi:10.1371/journal.pone.0026806)
Supplement: Table S5 — Matches between empirically derived T-RFs and predicted T-RFs from clone library 16S rRNA gene sequences, using the restriction endonucleases Hae III, Msp I and Rsa I. * = predicted T-RF size outside the sensitivity range of T-RFLP analysis (100–500 bp). (DOC) [file pone.0026806.s009.doc]

**Table S5.** Matches between empirically derived T-RFs and predicted T-RFs from clone library 16S rRNA gene sequences, using the restriction endonucleases *Hae*III, *Msp*I and *Rsa*I. * = predicted T-RF size outside the sensitivity range of T-RFLP analysis (100–500 bp).

|  |  | ***Hae*III** |  |  |  | ***Msp*I** |  |  |  | ***Rsa*I** |  |  |
| --- | --- | --- | --- | --- | --- | --- | --- | --- | --- | --- | --- | --- |
| **Clone** | **T-RFs** | **T-RF Range** | **Predicted** | **Match** | **T-RFs** | **T-RF Range** | **Predicted** | **Match** | **T-RFs** | **T-RF Range** | **Predicted** | **Match** |
| GOMB1 | - | **-** | 40 | * | 7 | 445.3 - 450.73 | 449 | Yes | - | - | 0 | * |
| GOMB2 | 3 | 291.06 - 292.70 | 292 | Yes | 3 | 489.05 - 491.22 | 492 | Yes | 5 | 420.00 - 425.81 | 423 | Yes |
| GOMB3 | - | **-** | 118 | No | - | - | 151 | No | - | - | 26 | * |
| GOMB4 | 2 | 407.56 - 408.23 | 411 | Yes | - | - | 544 | * | 2 | 313.13 - 314.41 | 314 | Yes |
| GOMB5 | - | **-** | 381 | No | - | - | 0 | * | - | - | 59 | * |
| GOMB6 | - | **-** | 310 | No | 1 | 166.91 | 167 | Yes | 1 | 440.26 | 439 | Yes |
| GOMB7 | - | **-** | 40 | * | - | - | 96 | * | 1 | 293.21 | 294 | Yes |
| GOMB8 | 2 | 194.03 - 194.44 | 194 | Yes | 9 | 436.32 - 443.34 | 440 | Yes | - | - | 119 | No |
| GOMB9 | - | **-** | 40 | * | 2 | 158.55 - 160.00 | 159 | Yes | 5 | 428.33 - 432.70 | 431 | Yes |
| GOMB10 | 2 | 337.52 - 338.39 | 338 | Yes | - | - | 97 | * | - | - | 0 | * |
| GOMB11 | 1 | 408.23 | 412 | Yes | - | - | 94 | * | 3 | 314.41 - 316.34 | 315 | Yes |
| GOMB12 | 1 | 208.85 | 208 | Yes | - | - | 510 | * | - | - | 0 | * |
| GOMB13 | - | - | 0 | * | - | - | 513 | * | - | - | 59 | * |
| GOMB14 | 4 | 227.76 - 230.08 | 229 | Yes | 9 | 437.37 - 444.28 | 441 | Yes | - | - | 26 | * |
| GOMB15 | - | - | 40 | * | - | - | 96 | * | - | - | 0 | * |
| GOMB16 | 2 | 337.52 - 338.39 | 338 | Yes | - | - | 509 | * | - | - | 0 | * |
| GOMB17 | - | - | 382 | No | - | - | 498 | No | - | - | 59 | * |
| GOMB18 | 3 | 191.2 - 192.89 | 192 | Yes | - | - | 81 | * | - | - | 0 | * |
| GOMB19 | 4 | 227.76 - 230.08 | 229 | Yes | 1 | 491.22 | 495 | Yes | - | - | 59 | * |
| GOMB20 | - | - | 40 | * | - | - | 96 | * | 4 | 315.72 - 318.19 | 317 | Yes |
| GOMB21 | 3 | 194.03 - 195.24 | 195 | Yes | 9 | 437.37 - 444.28 | 441 | Yes | - | - | 26 | * |
| GOMB22 | - | - | 40 | * | 7 | 445.3 - 450.73 | 449 | Yes | - | - | 0 | * |
| GOMB23 | - | - | 40 | * | - | - | 96 | * | 4 | 446.37 - 453.00 | 449 | Yes |
| GOMB24 | 2 | 194.03 - 194.44 | 194 | Yes | 9 | 436.32 - 443.34 | 440 | Yes | - | - | 119 | No |
| GOMB25 | - | - | 382 | No | - | - | 498 | No | - | - | 59 | * |
| GOMB26 | - | - | 0 | * | 3 | 468.33 - 470.41 | 471 | Yes | - | - | 84 | * |
| GOMB27 | - | - | 40 | * | - | - | 497 | No | 7 | 424.64 - 431.95 | 428 | Yes |
| GOMB28 | 4 | 403.91 - 408.23 | 407 | Yes | - | - | 0 | * | - | - | 85 | * |
| GOMB29 | - | - | 40 | * | - | - | 85 | * | 7 | 424.64 - 431.95 | 428 | Yes |
| GOMB30 | 1 | 274 | 274 | Yes | - | - | 271 | No | 3 | 440.26 - 447.86 | 444 | Yes |
| GOMB31 | - | - | 40 | * | 6 | 446.05 - 450.73 | 450 | Yes | - | - | 0 | * |
| GOMB32 | - | - | 118 | No | 9 | 437.37 - 444.28 | 441 | Yes | - | - | 72 | * |
| GOMB33 | 2 | 191.2 - 191.73 | 191 | Yes | - | - | 182 | No | - | - | 0 | * |
| GOMB34 | - | - | 310 | No | - | - | 456 | No | 1 | 440.26 | 439 | Yes |
| GOMB35 | 2 | 202.61 - 204.41 | 204 | Yes | - | - | 507 | * | - | - | 582 | * |
| GOMB36 | - | - | 241 | No | 2 | 449.46 - 450.73 | 453 | Yes | - | - | 69 | * |
| GOMB37 | 2 | 195.24 - 196.02 | 196 | Yes | - | - | 499 | No | - | - | 0 | * |
| GOMB38 | 1 | 257.64 | 257 | Yes | 2 | 170.33 - 171.26 | 171 | Yes | - | - | 138 | No |
| GOMB39 | 4 | 228.85 - 231.32 | 230 | Yes | 1 | 313.64 | 314 | Yes | - | - | 484 | No |
| GOMB41 | 3 | 194.03 - 195.24 | 195 | Yes | 9 | 437.37 - 444.28 | 441 | Yes | - | - | 26 | * |
| GOMB42 | - | - | 139 | No | 3 | 489.05 - 491.22 | 492 | Yes | 5 | 420.00 - 425.81 | 423 | Yes |
| GOMB43 | 3 | 329.57 - 332.27 | 331 | Yes | - | - | 477 | No | - | - | 460 | No |
| GOMB44 | - | - | 167 | No | - | - | 458 | No | - | - | 533 | * |
| GOMB47 | 2 | 202.61 - 204.41 | 204 | Yes | - | - | 294 | No | - | - | 490 | No |
| GOMB48 | - | - | 66 | * | 3 | 448.58 - 450.73 | 452 | Yes | - | - | 131 | No |
| GOMB49 | 1 | 290.1 | 289 | Yes | - | - | 547 | * | 4 | 315.72 - 318.19 | 317 | Yes |
| GOMB50 | - | - | 0 | * | 2 | 170.33 - 171.26 | 171 | Yes | - | - | 513 | * |
| GOMB68 | 2 | 323.36 - 324.48 | 324 | Yes | 3 | - | 146 | No | - | - | 0 | * |
| GOMB69 | - | - | 382 | No | - | - | 498 | No | - | - | 59 | * |
| GOMB70 | 1 | 220.37 | 220 | Yes | 4 | 430.93 - 433.44 | 431 | Yes | - | - | 474 | No |
| GOMB71 | 1 | 261.96 | 261 | Yes | - | - | 599 | * | 1 | 469.45 | 471 | Yes |
| GOMB72 | - | - | 240 | No | - | - | 90 | * | - | - | 0 | * |
| GOMB73 | - | - | 288 | No | - | - | 163 | No | 1 | 234.59 | 234 | Yes |
| GOMB74 | 1 | 292.70 | 294 | Yes | - | - | 164 | No | 1 | 115.07 | 116 | Yes |
| GOMB75 | - | - | 382 | No | - | - | 498 | No | - | - | 59 | * |
| GOMB76 | 1 | 192.89 | 193 | Yes | - | - | 78 | * | - | - | 0 | * |
| GOMB77 | - | - | 288 | No | - | - | 163 | No | 3 | 453.00 - 455.09 | 457 | Yes |
| GOMB78 | 2 | 416.33 - 417.75 | 415 | Yes | - | - | 97 | * | - | - | 120 | No |
| GOMB79 | 1 | 261.96 | 261 | Yes | - | - | 599 | * | 1 | 469.45 | 471 | Yes |
| GOMB80 | - | - | 265 | No | - | - | 68 | * | - | - | 204 | No |
| GOMB81 | - | - | 40 | * | - | - | 197 | No | - | - | 564 | * |
| GOMB82 | - | - | 0 | * | - | - | 498 | No | - | - | 59 | * |
| GOMB83 | - | - | 382 | No | - | - | 498 | No | - | - | 59 | * |
| GOMB84 | 3 | 204.41 - 206.34 | 205 | Yes | 1 | 491.22 | 495 | Yes | - | - | 74 | * |
| GOMB85 | 2 | 258.67 - 259.45 | 260 | Yes | - | - | 497 | No | - | - | 480 | No |
| GOMB86 | 3 | 232.61 - 234.56 | 234 | Yes | - | - | 599 | * | 1 | 469.45 | 471 | Yes |
| GOMB87 | 3 | 201.69 - 204.41 | 203 | Yes | 1 | 145.00 | 144 | Yes | - | - | 476 | No |
| GOMB88 | - | - | 466 | No | - | - | 94 | * | 1 | 133.18 | 133 | Yes |
| GOMB89 | - | - | 40 | * | - | - | 507 | * | 1 | 440.26 | 438 | Yes |
| GOMB90 | 1 | 208.85 | 208 | Yes | 3 | 145.00 - 146.59 | 146 | Yes | 1 | 455.09 | 458 | Yes |
| GOMB91 | - | - | 295 | No | - | - | 165 | No | - | - | 120 | No |
| GOMB92 | - | - | 0 | * | - | - | 505 | * | - | - | 59 | * |
| GOMB93 | - | - | 40 | * | - | - | 176 | No | - | - | 131 | No |
| GOMB94 | - | - | 40 | * | - | - | 199 | No | - | - | 566 | * |
| GOMB95 | 2 | 258.67 - 259.45 | 260 | Yes | - | - | 497 | No | - | - | 0 | * |
| GOMB96 | - | - | 78 | * | - | - | 325 | No | - | - | 0 | * |
| GOMB97 | - | - | 40 | * | 9 | 436.32 - 443.34 | 440 | Yes | - | - | 0 | * |
| GOMB98 | - | - | 0 | * | - | - | 92 | * | 2 | 114.22 - 115.07 | 115 | Yes |
| GOMB99 | 3 | 257.64 - 259.45 | 258 | Yes | 1 | 491.22 | 495 | Yes | - | - | 0 | * |
| GOMB100 | 3 | 226.87 - 228.85 | 228 | Yes | 9 | 436.32 - 443.34 | 440 | Yes | - | - | 0 | * |
| GOMB101 | - | - | 382 | No | - | - | 498 | No | - | - | 59 | * |
| GOMB102 | 3 | 329.57 - 332.27 | 331 | Yes | - | - | 477 | No | - | - | 460 | No |
| GOMB103 | 1 | 292.70 | 294 | Yes | 9 | 436.32 - 443.34 | 440 | Yes | - | - | 26 | * |
| GOMB104 | - | - | 40 | * | - | - | 92 | * | 3 | 310.97 - 313.13 | 312 | Yes |
| GOMB105 | 4 | 227.76 - 230.08 | 229 | Yes | 9 | 437.37 - 444.28 | 441 | Yes | - | - | 26 | * |
| GOMB106 | - | - | 40 | * | 8 | 435.12 - 440.96 | 438 | Yes | - | - | 0 | * |
| GOMB107 | 2 | 337.52 - 338.39 | 338 | Yes | 2 | 158.55 - 160.00 | 159 | Yes | - | - | 481 | No |
| GOMB108 | 1 | 292.70 | 294 | Yes | - | - | 151 | No | 5 | 420.00 - 425.81 | 423 | Yes |
| GOMB109 | 2 | 189.23 - 189.88 | 189 | Yes | 1 | 141.69 | 141 | Yes | - | - | 0 | * |
| GOMB110 | - | - | 40 | * | 4 | 485.41 - 491.22 | 489 | Yes | - | - | 564 | * |
| GOMB111 | - | - | 40 | * | - | - | 507 | * | - | - | 0 | * |
| GOMB112 | 2 | 194.03 - 194.44 | 194 | Yes | - | - | 164 | No | - | - | 0 | * |
| GOMB113 | 2 | 194.03 - 194.44 | 194 | Yes | - | - | 164 | No | - | - | 0 | * |
| GOMB114 | 2 | 195.24 - 196.02 | 196 | Yes | - | - | 507 | * | - | - | 463 | No |
| GOMB115 | 2 | 223.74 - 224.88 | 224 | Yes | 1 | 491.22 | 495 | Yes | - | - | 0 | * |
| GOMB116 | - | - | 40 | * | - | - | 210 | No | 3 | 314.41 - 316.34 | 315 | Yes |
| GOMB117 | 1 | 313.82 | 313 | Yes | 1 | 313.64 | 315 | Yes | - | - | 85 | * |
| GOMB118 | 3 | 204.41 - 206.34 | 205 | Yes | 1 | 491.22 | 495 | Yes | - | - | 570 | * |
| GOMB119 | 1 | 313.82 | 313 | Yes | 1 | 313.64 | 315 | Yes | - | - | 85 | * |
| GOMB120 | - | - | 118 | No | 9 | 437.37 - 444.28 | 441 | Yes | - | - | 26 | * |
| GOMB121 | - | - | 295 | No | - | - | 151 | No | 5 | 420.00 - 425.81 | 424 | Yes |
| GOMB122 | 2 | 189.23 - 189.88 | 189 | Yes | 1 | 141.69 | 141 | Yes | - | - | 0 | * |
| GOMB123 | 1 | 292.70 | 294 | Yes | 9 | 436.32 - 443.34 | 440 | Yes | - | - | 26 | * |
| GOMB124 | 1 | 313.82 | 313 | Yes | 1 | 313.64 | 315 | Yes | - | - | 0 | * |
| GOMB125 | 2 | 189.23 - 189.88 | 189 | Yes | 3 | 489.05 - 491.22 | 490 | Yes | - | - | 0 | * |
| GOMB126 | 1 | 251.2 | 250 | Yes | - | - | 496 | No | - | - | 0 | * |
| GOMB127 | - | - | 66 | * | 3 | 448.58 - 450.73 | 452 | Yes | - | - | 131 | No |
| GOMB128 | 3 | 232.07 - 233.84 | 233 | Yes | - | - | 153 | No | - | - | 488 | No |
| GOMB129 | 3 | 194.03 - 195.24 | 195 | Yes | 9 | 437.37 - 444.28 | 441 | Yes | 5 | 420.00 - 425.81 | 424 | Yes |
| GOMB130 | 3 | 406.26 - 408.23 | 410 | Yes | - | - | 92 | * | 2 | 114.22 - 115.07 | 115 | Yes |
| GOMB131 | - | - | 310 | No | - | - | 456 | No | - | - | 65 | * |
| GOMB132 | - | - | 66 | * | 3 | 448.58 - 450.73 | 452 | Yes | - | - | 131 | No |
| GOMB133 | - | - | 288 | No | - | - | 210 | No | 3 | 314.41 - 316.34 | 315 | Yes |
| GOMB134 | - | - | 241 | No | - | - | 163 | No | - | - | 85 | * |
| GOMB135 | 2 | 195.24 - 196.02 | 196 | Yes | - | - | 507 | Yes | 1 | 440.26 | 438 | Yes |
| GOMB136 | - | - | 237 | No | - | - | 133 | No | 1 | 455.09 | 459 | Yes |
| GOMB137 | 1 | 192.89 | 193 | Yes | - | - | 176 | No | - | - | 215 | No |
| GOMB138 | 1 | 285.88 | 285 | Yes | - | - | 94 | * | - | - | 110 | No |
| GOMB139 | 2 | 319.63 - 320.86 | 320 | Yes | 1 | 141.69 | 141 | Yes | - | - | 69 | * |
| GOMB140 | 4 | 227.76 - 230.08 | 229 | Yes | 9 | 437.37 - 444.28 | 441 | Yes | 5 | 420.00 - 425.81 | 424 | Yes |
| GOMB141 | 1 | 257.64 | 257 | Yes | 3 | 468.33 - 470.41 | 469 | Yes | 2 | 310.97 - 311.91 | 311 | Yes |
| GOMB142 | - | - | 40 | * | - | - | 507 | * | - | - | 0 | * |
| GOMB143 | - | - | 40 | * | 4 | 485.41 - 491.22 | 489 | Yes | - | - | 564 | * |
| GOMB144 | - | - | 172 | No | - | - | 0 | * | - | - | 59 | * |
| GOMB145 | - | - | 40 | * | 4 | 485.41 - 491.22 | 489 | Yes | - | - | 564 | * |
| GOMB146 | 1 | 292.70 | 294 | Yes | 9 | 436.32 - 443.34 | 440 | Yes | - | - | 26 | * |
| GOMB147 | - | - | 163 | No | 2 | 449.46 - 450.73 | 453 | Yes | - | - | 251 | No |
| GOMB148 | - | - | 295 | No | - | - | 151 | No | - | - | 0 | * |
| GOMB149 | - | - | 40 | * | - | - | 92 | * | 3 | 310.97 - 313.13 | 312 | Yes |
| GOMB150 | 2 | 223.74 - 224.88 | 224 | Yes | 1 | 491.22 | 495 | Yes | - | - | 0 | * |
| GOMB151 | - | - | 40 | * | - | - | 94 | * | 3 | 311.91 - 314.41 | 313 | Yes |
| GOMB152 | 3 | 204.41 - 206.34 | 205 | Yes | - | - | 496 | No | - | - | 0 | * |
| GOMB153 | - | - | 40 | * | - | - | 92 | * | 3 | 310.97 - 313.13 | 312 | Yes |
| GOMB154 | - | - | 40 | * | - | - | 97 | * | 2 | 317.61 - 318.19 | 319 | Yes |
| GOMB155 | 1 | 292.70 | 294 | Yes | - | - | 151 | No | - | - | 26 | * |
| GOMB156 | 2 | 407.56 - 408.23 | 411 | Yes | - | - | 544 | * | 2 | 313.13 - 314.41 | 314 | Yes |
| GOMB157 | - | - | 40 | * | 1 | 141.69 | 141 | Yes | - | - | 0 | * |
| GOMB158 | - | - | 66 | * | - | - | 68 | * | - | - | 0 | * |
| GOMB159 | 2 | 261.96 - 262.82 | 262 | Yes | 1 | 147.65 | 148 | Yes | - | - | 0 | * |
