# Supplementary material for: Phylogenetic Diversity, Host-Specificity and Community Profiling of Sponge-Associated Bacteria in the Northern Gulf of Mexico
Source: PLoS One. 2011 Nov 2;6(11):e26806. doi: 10.1371/journal.pone.0026806 (PMC3206846; doi:10.1371/journal.pone.0026806)
Supplement: Table S6 — Individual T-RFs recovered using the enzyme Hae III and matching 16S rRNA gene sequence OTUs from clone library analyses. (DOC) [file pone.0026806.s010.doc]

**Table S6.** Individual T-RFs recovered using the enzyme *Hae*III and matching 16S rRNA gene sequence OTUs from clone library analyses.

| **T-RF (bp)** | **Match** | **Bacteria Division** |
| --- | --- | --- |
| 189.23 | GOMB-109, 122, 125 | Gamma-proteobacteria |
| 189.88 | GOMB-109, 122, 125 | Gamma-proteobacteria |
| 191.20 | GOMB-18, 33 | Gamma-proteobacteria |
| 191.73 | GOMB-18, 33 | Gamma-proteobacteria |
| 192.89 | GOMB-18, 76, 137 | Alpha/Gamma-proteobacteria |
| 194.03 | GOMB-8, 21, 24, 41, 112, 113, 129 | Alpha-proteobacteria |
| 194.44 | GOMB-8, 21, 24, 41, 112, 113, 129 | Alpha-proteobacteria |
| 195.24 | GOMB-21, 37, 41, 114, 129, 135 | Alpha/Gamma-proteobacteria |
| 196.02 | GOMB-37, 114, 135 | Gamma-proteobacteria |
| 201.69 | GOMB-87 | Beta-proteobacteria |
| 202.61 | GOMB-35, 47, 87 | Beta/Gamma-proteobacteria |
| 204.41 | GOMB-35, 47, 84, 87, 118, 152 | Beta/Gamma-proteobacteria |
| 205.57 | GOMB-84, 118, 152 | Gamma-proteobacteria |
| 206.34 | GOMB-84, 118, 152 | Gamma-proteobacteria |
| 208.85 | GOMB-12, 90 | Firmicutes/Gamma-proteobacteria |
| 220.37 | GOMB-70 | Beta-proteobacteria |
| 223.74 | GOMB-115, 150 | Beta/Gamma-proteobacteria |
| 224.88 | GOMB-115, 150 | Beta/Gamma-proteobacteria |
| 226.87 | GOMB-100 | Alpha-proteobacteria |
| 227.76 | GOMB-14, 19, 100, 105, 140 | Cyanobacteria/Alpha-proteobacteria |
| 228.85 | GOMB-14, 19, 39, 100, 105, 140 | Cyanobacteria/Alpha/Beta-proteobacteria |
| 229.53 | GOMB-14, 19, 39, 105, 140 | Cyanobacteria/Alpha/Beta-proteobacteria |
| 230.08 | GOMB-14, 19, 39, 105, 140 | Cyanobacteria/Alpha/Beta-proteobacteria |
| 231.32 | GOMB-39 | Gamma-proteobacteria |
| 232.07 | GOMB-128 | Firmicutes |
| 232.61 | GOMB-86, 128 | Firmicutes/Nitrospira |
| 233.84 | GOMB-86, 128 | Firmicutes/Nitrospira |
| 234.56 | GOMB-86 | Nitrospira |
| 251.20 | GOMB-126 | Alpha-proteobacteria |
| 257.64 | GOMB-38, 99, 141 | Planctomycetes/Gamma-proteobacteria |
| 258.67 | GOMB-85, 95, 99 | Planctomycetes/Gamma-proteobacteria |
| 259.45 | GOMB-85, 95, 99 | Planctomycetes/Gamma-proteobacteria |
| 261.96 | GOMB-71, 79, 159 | Nitrospira/Gamma-proteobacteria |
| 262.82 | GOMB-159 | Gamma-proteobacteria |
| 274.00 | GOMB-30 | Delta-proteobacteria |
| 285.88 | GOMB-138 | Bacteroidetes |
| 290.10 | GOMB-49 | Bacteroidetes |
| 291.06 | GOMB-2 | Cyanobacteria |
| 292.02 | GOMB-2 | Cyanobacteria |
| 292.70 | GOMB-2, 74, 103, 108, 123, 146, 155 | Cyanobacteria/Alpha-proteobacteria |
| 313.82 | GOMB-117, 119, 124 | Gamma-proteobacteria |
| 319.63 | GOMB-139 | Gamma-proteobacteria |
| 320.86 | GOMB-139 | Gamma-proteobacteria |
| 323.36 | GOMB-68 | Gamma-proteobacteria |
| 324.48 | GOMB-68 | Gamma-proteobacteria |
| 329.57 | GOMB-43, 102 | Firmicutes/Actinobacteria |
| 331.25 | GOMB-43, 102 | Firmicutes/Actinobacteria |
| 332.27 | GOMB-43, 102 | Firmicutes/Actinobacteria |
| 337.52 | GOMB-10, 16, 107 | Gamma-proteobacteria |
| 338.39 | GOMB-10, 16, 107 | Gamma-proteobacteria |
| 403.91 | GOMB-28 | Delta-proteobacteria |
| 406.26 | GOMB-28, 130 | Bacteroidetes/Delta-proteobacteria |
| 407.56 | GOMB-4, 28, 130, 156 | Bacteroidetes/Delta-proteobacteria |
| 408.23 | GOMB-4, 11, 28, 130, 156 | Bacteroidetes/Delta-proteobacteria |
| 416.33 | GOMB-78 | Bacteroidetes |
| 417.75 | GOMB-78 | Bacteroidetes |
