# Supplementary material for: Phylogenetic Diversity, Host-Specificity and Community Profiling of Sponge-Associated Bacteria in the Northern Gulf of Mexico
Source: PLoS One. 2011 Nov 2;6(11):e26806. doi: 10.1371/journal.pone.0026806 (PMC3206846; doi:10.1371/journal.pone.0026806)
Supplement: Table S7 — Individual T-RFs recovered using the enzyme Msp I and matching 16S rRNA gene sequence OTUs from clone library analyses. (DOC) [file pone.0026806.s011.doc]

**Table S7.** Individual T-RFs recovered using the enzyme *Msp*I and matching 16S rRNA gene sequence OTUs from clone library analyses.

| **T-RF (bp)** | **Match** | **Bacteria Division** |
| --- | --- | --- |
| 141.69 | GOMB-109, 122, 139, 157 | Gamma-proteobacteria |
| 145.00 | GOMB-68, 87, 90 | Beta/Gamma-proteobacteria |
| 145.88 | GOMB-68, 90 | Gamma-proteobacteria |
| 146.59 | GOMB-68, 90 | Gamma-proteobacteria |
| 147.65 | GOMB-159 | Gamma-proteobacteria |
| 158.55 | GOMB-9, 107 | Alpha-proteobacteria |
| 160.00 | GOMB-9, 107 | Alpha-proteobacteria |
| 166.91 | GOMB-6 | Alpha-proteobacteria |
| 170.33 | GOMB-38, 50 | Planctomycetes/Verrucomicrobia |
| 171.26 | GOMB-38, 50 | Planctomycetes/Verrucomicrobia |
| 313.64 | GOMB-39, 117, 119, 124 | Gamma-proteobacteria |
| 430.93 | GOMB-70 | Beta-proteobacteria |
| 431.82 | GOMB-70 | Beta-proteobacteria |
| 432.58 | GOMB-70 | Beta-proteobacteria |
| 433.44 | GOMB-70 | Beta-proteobacteria |
| 435.12 | GOMB-106 | Alpha-proteobacteria |
| 436.32 | GOMB-8, 24, 97, 100, 103, 106, 123, 146 | Alpha-proteobacteria |
| 437.37 | GOMB-8, 14, 21, 24, 32, 41, 97, 100, 103, 105, 106, 120, 123, 129, 140, 146 | Alpha-proteobacteria |
| 437.93 | GOMB-8, 14, 21, 24, 32, 41, 97, 100, 103, 105, 106, 120, 123, 129, 140, 146 | Alpha-proteobacteria |
| 438.77 | GOMB-8, 14, 21, 24, 32, 41, 97, 100, 103, 105, 106, 120, 123, 129, 140, 146 | Alpha-proteobacteria |
| 439.73 | GOMB-8, 14, 21, 24, 32, 41, 97, 100, 103, 105, 106, 120, 123, 129, 140, 146 | Alpha-proteobacteria |
| 440.46 | GOMB-8, 14, 21, 24, 32, 41, 97, 100, 103, 105, 106, 120, 123, 129, 140, 146 | Alpha-proteobacteria |
| 440.96 | GOMB-8, 14, 21, 24, 32, 41, 97, 100, 103, 105, 106, 120, 123, 129, 140, 146 | Alpha-proteobacteria |
| 442.39 | GOMB-8, 14, 21, 24, 32, 41, 97, 100, 103, 105, 106, 120, 123, 129, 140, 146 | Alpha-proteobacteria |
| 443.34 | GOMB-8, 14, 21, 24, 32, 41, 97, 100, 103, 105, 106, 120, 123, 129, 140, 146 | Alpha-proteobacteria |
| 444.28 | GOMB-14, 21, 32, 41, 105, 120, 129, 140 | Alpha-proteobacteria |
| 445.30 | GOMB-1, 22 | Alpha-proteobacteria |
| 446.05 | GOMB-1, 22 | Alpha-proteobacteria |
| 446.79 | GOMB-1, 22, 31 | Alpha-proteobacteria |
| 447.91 | GOMB-1, 22, 31 | Alpha-proteobacteria |
| 448.58 | GOMB-1, 22, 31, 48, 127, 132 | Alpha-proteobacteria |
| 449.46 | GOMB-1, 22, 31, 36, 48, 127, 132 | Alpha/Gamma-proteobacteria |
| 450.73 | GOMB-1, 22, 31, 36, 48, 127, 132 | Alpha/Gamma-proteobacteria |
| 468.33 | GOMB-26, 141 | Delta/Epsilon-proteobacteria |
| 469.28 | GOMB-26, 141 | Delta/Epsilon-proteobacteria |
| 470.41 | GOMB-26, 141 | Delta/Epsilon-proteobacteria |
| 485.41 | GOMB-110, 143, 145 | Gamma-proteobacteria |
| 489.05 | GOMB-2, 42, 110, 125, 143, 145 | Cyano/Gamma-proteobacteria |
| 490.44 | GOMB-2, 42, 110, 125, 143, 145 | Cyano/Gamma-proteobacteria |
| 491.22 | GOMB-2, 42, 84, 99, 110, 115, 118, 125, 143, 145, 150 | Cyano/Beta/Gamma-proteobacteria |
