# Supplementary material for: Phylogenetic Diversity, Host-Specificity and Community Profiling of Sponge-Associated Bacteria in the Northern Gulf of Mexico
Source: PLoS One. 2011 Nov 2;6(11):e26806. doi: 10.1371/journal.pone.0026806 (PMC3206846; doi:10.1371/journal.pone.0026806)
Supplement: Table S8 — Individual T-RFs recovered using the enzyme Rsa I and matching 16S rRNA gene sequence OTUs from clone library analyses. (DOC) [file pone.0026806.s012.doc]

**Table S8.** Individual T-RFs recovered using the enzyme *Rsa*I and matching 16S rRNA gene sequence OTUs from clone library analyses.

| **T-RF (bp)** | **Match** | **Bacteria Division** |
| --- | --- | --- |
| 114.22 | GOMB-98, 130 | Bacteroidetes |
| 115.07 | GOMB-74, 98, 130 | Bacteroidetes/Alpha-proteobacteria |
| 133.18 | GOMB-88 | Bacteroidetes |
| 234.59 | GOMB-73 | Gamma-proteobacteria |
| 293.21 | GOMB-7 | Gamma-proteobacteria |
| 310.97 | GOMB-104, 141, 149, 153 | Bacteroidetes/Epsilon-proteobacteria |
| 311.91 | GOMB-104, 141, 149, 153 | Bacteroidetes/Epsilon-proteobacteria |
| 313.13 | GOMB-4, 104, 149, 151, 153, 156 | Bacteroidetes |
| 314.41 | GOMB-4, 11, 116, 133, 151, 156 | Bacteroidetes |
| 315.72 | GOMB-11, 20, 49, 116, 133 | Bacteroidetes |
| 316.34 | GOMB-11, 20, 49, 116, 133 | Bacteroidetes |
| 317.61 | GOMB-20, 49, 154 | Bacteroidetes |
| 318.19 | GOMB-20, 49, 154 | Bacteroidetes |
| 420.00 | GOMB-2, 42, 108, 121, 129, 140 | Cyanobacteria/Alpha-proteobacteria |
| 420.88 | GOMB-2, 42, 108, 121, 129, 140 | Cyanobacteria/Alpha-proteobacteria |
| 424.64 | GOMB-2, 27, 29, 42, 108, 121, 129, 140 | Cyanobacteria/Alpha/Gamma-proteobacteria |
| 425.27 | GOMB-2, 27, 29, 42, 108, 121, 129, 140 | Cyanobacteria/Alpha/Gamma-proteobacteria |
| 425.81 | GOMB-2, 27, 29, 42, 108, 121, 129, 140 | Cyanobacteria/Alpha/Gamma-proteobacteria |
| 428.33 | GOMB-9, 27, 29 | Alpha/Gamma-proteobacteria |
| 429.94 | GOMB-9, 27, 29 | Alpha/Gamma-proteobacteria |
| 430.99 | GOMB-9, 27, 29 | Alpha/Gamma-proteobacteria |
| 431.95 | GOMB-9, 27, 29 | Alpha/Gamma-proteobacteria |
| 432.70 | GOMB-9 | Alpha-proteobacteria |
| 440.26 | GOMB-6, 30, 34, 89, 135 | Alpha/Gamma/Delta-proteobacteria |
| 446.37 | GOMB-23, 30 | Alpha/Delta-proteobacteria |
| 447.86 | GOMB-23, 30 | Alpha/Delta-proteobacteria |
| 448.91 | GOMB-23 | Alpha-proteobacteria |
| 453.00 | GOMB-23, 77 | Alpha/Delta-proteobacteria |
| 453.91 | GOMB-77 | Delta-proteobacteria |
| 455.09 | GOMB-77, 90 | Firmicutes/Delta-proteobacteria |
| 469.45 | GOMB-71, 79, 86 | Nitrospira |
