# Supplementary material for: Phylogenetic Diversity, Host-Specificity and Community Profiling of Sponge-Associated Bacteria in the Northern Gulf of Mexico
Source: PLoS One. 2011 Nov 2;6(11):e26806. doi: 10.1371/journal.pone.0026806 (PMC3206846; doi:10.1371/journal.pone.0026806)
Supplement: Table S9 — Sample number, platform number and GPS coordinates of sponge, tunicate and seawater samples collected from drilling platform pilings in the northern Gulf of Mexico. (DOC) [file pone.0026806.s013.doc]

**Table S9**. Sample number, platform number and GPS coordinates of sponge, tunicate and seawater samples collected from drilling platform pilings in the northern Gulf of Mexico.

| Source | No. Samples | Platform | Date | Longitude | Latitude |
| --- | --- | --- | --- | --- | --- |
| *Hymeniacidon heliophila* | 3 | 870A | 11/3/2003 | N 30º08.271 | W 88º00.145 |
| *Hymeniacidon heliophila* | 3 | 115C | 11/3/2003 | N 30º11.353 | W 87º57.177 |
| *Hymeniacidon heliophila* | 3 | 114 | 11/3/2003 | N 30º11.376 | W 88º01.312 |
|  |  |  |  |  |  |
| *Haliclona tubifera* | 1 | 870A | 11/3/2003 | N 30º08.271’ | W 88º00.245’ |
| *Haliclona tubifera* | 1 | 115C | 8/14/2003 | N 30º11.353’ | W 87º57.177’ |
| *Haliclona tubifera* | 1 | 114 | 11/3/2003 | N 30º11.376’ | W 88º01.312’ |
|  |  |  |  |  |  |
| Ambient Seawater | 3 | 870A | 11/3/2003 | N 30º08.271’ | W 88º00.245’ |
| Ambient Seawater | 3 | 115C | 11/3/2003 | N 30º11.353’ | W 87º57.177’ |
| Ambient Seawater | 3 | 114 | 11/3/2003 | N 30º11.376’ | W 88º01.312’ |
|  |  |  |  |  |  |
| *Didemnum* sp. | 1 | 870A | 11/3/2003 | N 30º08.271’ | W 88º00.245’ |
| *Didemnum* sp. | 1 | 115C | 10/7/2002 | N 30º11.353’ | W 87º57.177’ |
| *Didemnum* sp. | 1 | 827 | 10/7/2002 | N 30º10.654’ | W 87º56.356’ |
|  |  |  |  |  |  |
| *Halichondria* sp. | 1 | 864 | 10/9/2002 | N 30º08.977’ | W 88º17.200’ |
| *Halichondria* sp. | 1 | 115C | 8/14/2002 | N 30º11.353’ | W 87º57.177’ |
